# Genome sequence of pacific abalone (*Haliotis discus hannai*): the first draft genome in family Haliotidae

**DOI:** 10.1093/gigascience/gix014

**Published:** 2017-03-07

**Authors:** Bo-Hye Nam, Woori Kwak, Young-Ok Kim, Dong-Gyun Kim, Hee Jeong Kong, Woo-Jin Kim, Jeong-Ha Kang, Jung Youn Park, Cheul Min An, Ji-Young Moon, Choul Ji Park, Jae Woong Yu, Joon Yoon, Minseok Seo, Kwondo Kim, Duk Kyung Kim, SaetByeol Lee, Samsun Sung, Chul Lee, Younhee Shin, Myunghee Jung, Byeong-Chul Kang, Ga-hee Shin, Sojeong Ka, Kelsey Caetano-Anolles, Seoae Cho, Heebal Kim

**Affiliations:** 1 Biotechnology Research Division, National Institute of Fisheries Science, Haean-ro 216, Gijang-eup, Gijang-gun, Busan 619–705, Korea; 2 Interdisciplinary Program in Bioinformatics, Seoul National University, Gwanak-ro 1, Gwanak-gu, Seoul 151–747, Republic of Korea; 3 C&K Genomics, Main Bldg. #420, SNU Research Park, Gwanak-ro 1, Gwanak-gu, Seoul 151–919, Republic of Korea; 4 Genetics and Breeding Research Center, National Institute of Fisheries Science, 81–9, Geojenamseo-ro, Dapo-ri, Nambu-myeon, Geoje-si, Gyeongsangnam-do 53334, Republic of Korea; 5 Research and Development Center, Insilicogen Inc., 13, Heungdeok 1-ro, Giheung-gu, Yongin-si , Gyeonggi-do 16954, Republic of Korea; 6 Animal Science and Biotechnology, Seoul National University, Gwanak-ro 1, Gwanak-gu, Seoul 151–747, Republic of Korea; 7 Department of Agricultural Biotechnology and Research Institute for Agriculture and Life Sciences, Seoul National University, Gwanak-ro 1, Gwanak-gu, Seoul 151–921, Republic of Korea

**Keywords:** Abalone genome, Halotidae, *Haliotis discus hannai*

## Abstract

**Background:** Abalones are large marine snails in the family Haliotidae and the genus Haliotis belonging to the class Gastropoda of the phylum Mollusca. The family Haliotidae contains only one genus, Haliotis, and this single genus is known to contain several species of abalone. With 18 additional subspecies, the most comprehensive treatment of Haliotidae considers 56 species valid [1]. Abalone is an economically important fishery and aquaculture animal that is considered a highly prized seafood delicacy. The total global supply of abalone has increased 5-fold since the 1970s and farm production increased explosively from 50 mt to 103 464 mt in the past 40 years. Additionally, researchers have recently focused on abalone given their reported tumor suppression effect. However, despite the valuable features of this marine animal, no genomic information is available for the Haliotidae family and related research is still limited. To construct the *H*. *discus hannai* genome, a total of 580-G base pairs using Illumina and Pacbio platforms were generated with 322-fold coverage based on the 1.8-Gb estimated genome size of *H*. *discus hannai* using flow cytometry. The final genome assembly consisted of 1.86 Gb with 35 450 scaffolds (>2 kb). GC content level was 40.51%, and the N50 length of assembled scaffolds was 211 kb. We identified 29 449 genes using Evidence Modeler based on the gene information from ab initio prediction, protein homology with known genes, and transcriptome evidence of RNA-seq. Here we present the first Haliotidae genome, *H*. *discus hannai*, with sequencing data, assembly, and gene annotation information. This will be helpful for resolving the lack of genomic information in the Haliotidae family as well as providing more opportunities for understanding gastropod evolution.

## Introduction

**Figure 1.**
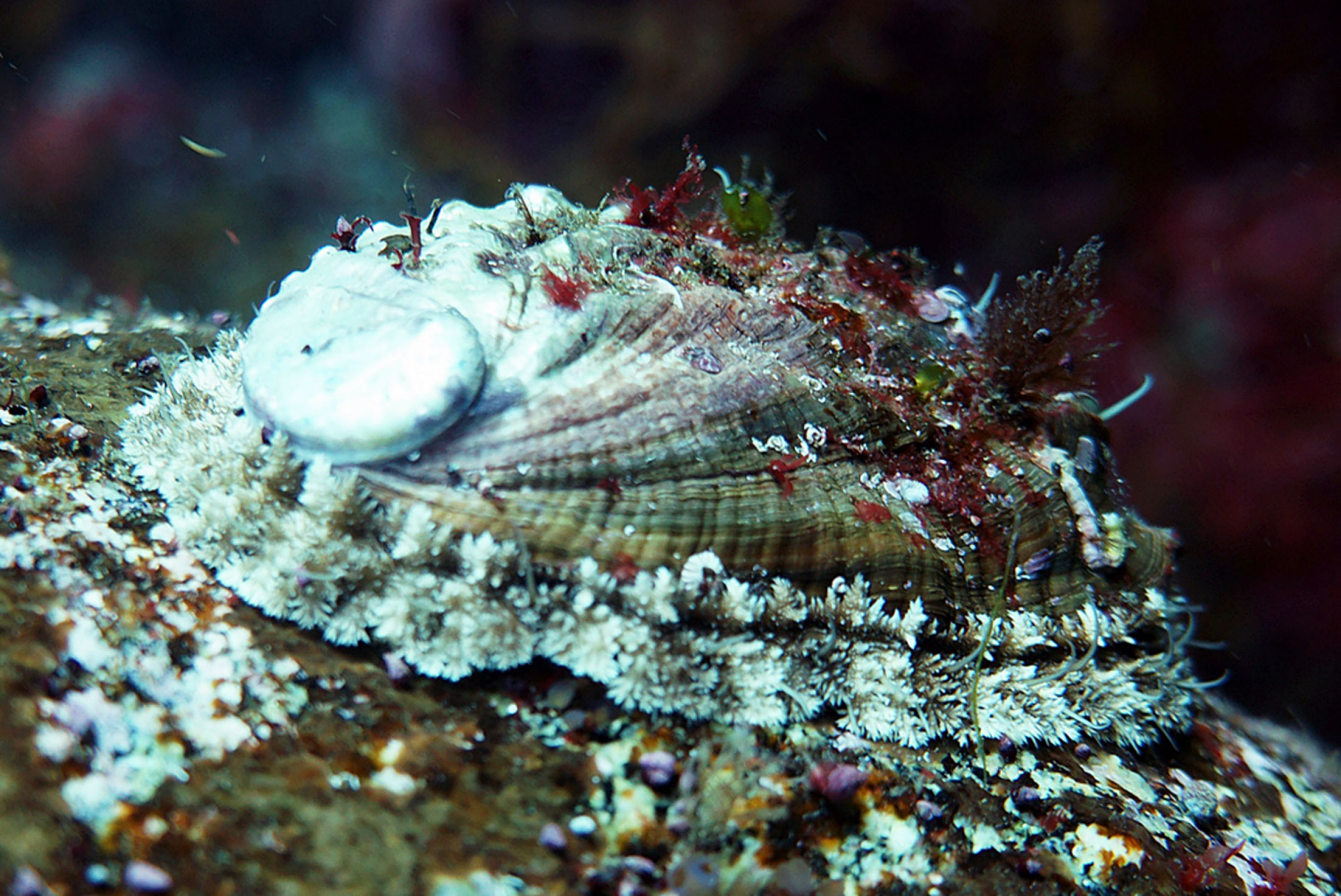
Example of a *H. discus hannai*, the pacific abalone.

Abalone is one of the most important marine gastropod mollusks that inhabits various coastal regions of the world. It is well known that abalone habitation impacts algal communications connected with the reef ecosystem, so they are often utilized for ecological research [2]. Among many abalone species, *Haliotis discus hannai* is a widely used ingredient in East Asian cuisine and is a valuable food resource due to its richness in protein and other nutrients (Fig. 1) [3, 4]. It is considered an important fishery industry animal. The total global supply of abalone has increased 5-fold since the 1970s. To prevent indiscreetly fishing abalones, legal landings from abalone fisheries have made fishery production decrease gradually from 19 720 mt to 7486 mt but have made farm productions increase explosively from 50 mt to 103 464 mt in the past 40 years [5]. Additionally, researchers have recently focused on *H. discus hannai* given its reported tumor suppression effect [6–8]. However, despite the valuable features of this marine animal, no genomic information is available. Therefore, the first draft genome in family Haliotidae has the potential to be utilized as a valuable resource for many researchers.

A single wild abalone (*H. discus hannai*) was collected from the brood stock at the Genetic and Breeding Research Center of the National Fisheries Research & Development Institute on Geoje Island, Korea for sampling. Hemolymph (10 ml) was withdrawn from the sole side foot muscle using a syringe. For genomic DNA extraction, hemocytes were harvested from fresh hemolymph by centrifugation at 3000 × rpm for 5 min at 4°C. Genomic DNA was extracted using a DNeasy Animal Mini Kit (Qiagen, Hilden, Germany). A total 39.38 μg of DNA was quantified using the standard procedure of Quant-iT PicoGreen dsDNA Assay Kit (Molecular Probes, Eugene, OR, USA) with Synergy HTX Multi-Mode Reader (Biotek, Winooski, VT, USA). Quality of DNA was also checked using ND-1000 spectrophotometer (Thermo Scientifc, Wilmington, DE, USA).

**Figure 2.**
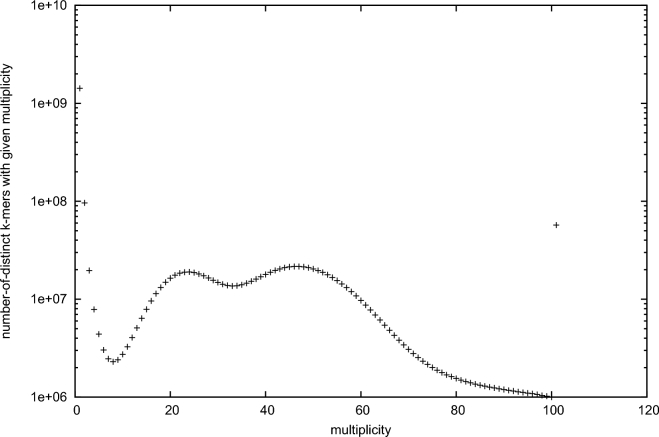
19-mer distribution of using jellyfish with 350-bp paired-end whole genome sequencing data.

For whole genome shotgun sequencing and draft genome assembly, we used multiple sequencing platforms (Illumina Hiseq2000, Nextseq500 and Pacbio RS II) with seven different libraries. First, two paired-end libraries with insert sizes of 250 and 350 bp were constructed using Illumina TruSeq DNA Sample Prep. Kit (Illumina, San Diego, CA, USA). Mate pair libraries with insert sizes of about 3, 5, 8, and 10 k were constructed for scaffolding process using Illumina Nextera mate-pair library construction protocol (Illumina). For high-quality genome assembly, long mate pair library with insert size over 40 kb is essential. We tried to construct a long mate pair library using 40 kb fosmid clone. However, efficiency of fosmid library construction was very low and we could not retain enough amount of clone. Therefore, Pacbio system was employed for final scaffolding process using long read. Pacbio long reads were generated using P6-C4 chemistry of Pacbio RS II system. Detailed information about the constructed library and generated sequencing data is provided in Table 1. Quality control process of generated raw data was conducted for downstream analysis. Quality of raw data was checked using FASTQC [9] and adapter sequences were removed via Trimmomatic [10], for paired-end libraries, and Nxtrim [11], for mate-pair libraries. K-mer frequency analysis of the abalone genome was conducted using a paired-end library with 350-bp insert size and the jellyfish [12] command-line program. The K-mer distribution of the paired-end library provides valuable information about the target genome. As a result, 19-mer distribution of *H. discus hannai* genome was generated (Fig. 2). Genome size estimation based on the 19-mer distribution was conducted through “Estimate genome size.pl” code (https://github.com/josephryan/estimate_genome_size.pl/wiki/Estimate-genome-size.pl”). The estimated genome size of *H. discus hannai* using 19-mer distribution was about 1.65 Gb. Based on the 19-mer distribution of paired-end reads, there was a second peak located in the half x-axis of the main peak. This result indicates that the *H. discus hannai* genome had high heterozygous genetic character or probable DNA contamination from other organisms. Therefore, before genome assembly, raw reads from Hiseq2000 and Nextseq500 paired-end and mate pairs were preprocessed by bacterial sequences, duplicates, and ambiguous nucleotides. To remove the contaminant sequence, clean reads without adapter and low quality bases were mapped to bacterial and ocean metagenome databases downloaded from NCBI by applying the default setting run (-s 0.8 –l 0.5) of clc_mapper (https://www.qiagenbioinformatics.com/). After that, duplicates and ambiguous nucleotides were filtered out using clc_remove_duplicates (https://www.qiagenbioinformatics.com/). The resulting high-quality sequences were used in subsequent assembly. Error correction and initial contig assembly was conducted using clc_assembler within the CLC Assembly Cell (https://www.qiagenbioinformatics.com/products/clc-assembly-cell/) software pipeline. Scaffolds were then built using the mate-pairs and Pacbio RS II reads sequentially by SSPACE [13] and PBJelly2 [14]. After scaffolding, we iteratively conducted gap filling process using Gapcloser [15] using -l 155 and -p 31 parameter option. Summary statistics for final assembly is provided in Table 2.

**Table 1.** Summary statistics of generated whole genome shotgun sequencing data.

Library name	Library type	Insert size	Platform	Read length	No. read	Total bp
250 bp	Paired-end	250	Nextseq500	150	876 529 480	131 440 418 087
350 bp	Paired-end	350	Hiseq2000	101	1 413 620 786	142 775 699 386
3 k	Mate-pair	3000	Nextseq500	150	580 064 464	85 689 154 056
5 k	Mate-pair	5000	Nextseq500	150	468 432 888	69 966 139 205
8 k	Mate-pair	8000	Nextseq500	150	335 132 792	50 109 845 012
10 k	Mate-pair	10 000	Nextseq500	150	569 376 096	85 080 237 236
20 k	P6-C4	20 000	Pacbio RS II	10 094		
(average)	1,573,020	15 879 626 978				
Total						580 941 119 960

**Table 2.** Summary statistics for the *H. discus hannai* draft genome (>2 kb).

Assembled genome	
Size (1n)	1.80 Gb
GC level	40.51%
No. scaffolds	35 450
N50 of scaffolds (bp)	211 346
N bases in scaffolds (%)	116 Mb (6.45%)
Longest (shortest) scaffolds (bp)	2 207 537 (2000)
Average scaffold length (bp)	50 870.65

**Figure 3.**
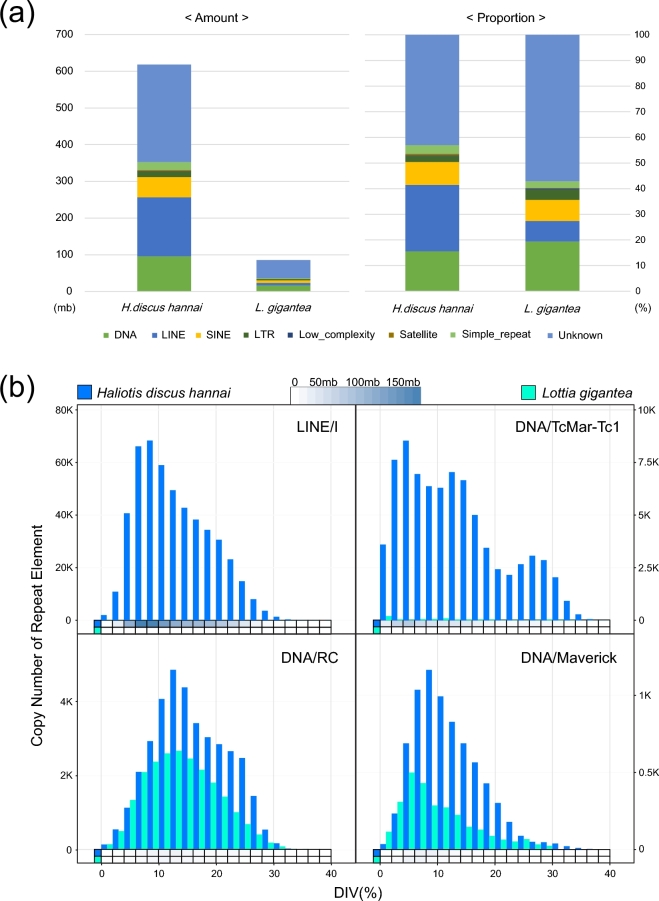
Repeat element information of *H. discus hannai* compared to *L. gigantean*. (**a**) Total amount and ratio of identified repeat element classified into eight classes (DNA, LINE, SINE, LTR, Low complexity, Satellite, Simple repeat, and Unknown) from each genome. (**b**) Distribution of gene copy number of the two highly possessed repeat elements in each genome based on the divergence. Heat maps indicate the total amount of repeat element divided into 20 levels based on the divergence.

Before conducting gene prediction using the assembled sequence, repeat elements were identified using RepeatMasker [16] with Repbase [17]. RepeatModeler, which includes RECON [18], RepeatScout [19], and TRF [20], was used to create a custom database of *H. discus hannai*. After custom library construction, RepeatMasker with RMBlast was used for each genome with ‘no_is’ option, using repeat libraries from RepeatModeler and Repbase. Identified mobile elements are summarized in Table 3. Identified repeat elements were parsed for identifying more detailed information using a perl code named “One code to find them all” [21] and Fig. S1 shows the proportion of each mobile element. The genome size of *H. discus hannai* was 1.86 Gb, and this is the biggest genome among known gastropods. It is 5.31 and 2.02 times larger than genomes size of *Lottia gigantea* (0.35 Gb) and *Aplysia californica* (0.92 Gb) in the same Gastropoda class. In animals, the increase of genome size is commonly driven by transposable element, and this is a known genetic adaption mechanism to stressful environments [22]. Therefore, we conducted comparative analysis of repeat element against *L. gignatea*, a similar marine gastropod with large genome size difference from that of *H. discus hannai*, to identify the reason for this large difference. Fig. 3a shows the amount and proportion of identified repeat element from two marine gastropods. The proportion of identified total repeat elements in *H. discus hannai* and *L. gigantea* is 30.76% and 22.25%, respectively. And the total amount of identified repeat elements in the *H. discus hannai* genome is almost six times larger than that of *L. gigantea* same as genome size. Such a linear relationship between genome size and the total proportion of repeat elements is consistent with a previous study [23]. The proportion, copy number, and divergence of each mobile element were identified and compared (Figs S2–6) for a deeper understanding of mobile elements in the two species. From the comparison, a notable finding has been observed on mobile elements: DNA transposable element, a Class II transposable element, exists in diverse forms in both species; however, retrotransposon element, a Class I transposable element, is much more abundant in *H. discus hannai* genome than in *L. gigantea* genome. Especially, the number of a non-LTR retrotransposon called LINE Element was exceptionally high. Fig. 3b illustrates the difference between the two species, using two signature mobile elements (*H. discus hannai*: LINE/I, DNA/TcMar-Tc1, *L. gigantea*: DNA/RC, DNA/Maverick) in each genome. DNA/RC and DNA/Maverick, two major mobile elements in *L. gigantea* genome, are observed in *H. discus* in somewhat similar distribution. On the other hand, the two signature mobile elements of *H. discus hannai* genome, LINE/I and DNA/TcMar-Tc1, are specifically abundant in *H. discus hannai* and seems to have expanded recently diverged compared to other elements. In sum, species specificity can be inferred from the distinctive patterns of repeat element expansion between the two species and the increased genome size of *H. discus hannai* may be associated with the non-LTR elements (especially LINE/I) contribution, in parallel to the human genome [23].

**Table 3.** Summary of identified repeat elements in the *Haliotis discus hannai* genome.

Repeat element	No. element	Length (%)
SINE	284 485	96 155 199 (5.11%)
LINE	700 245	160 387 248 (8.53%)
LTR element	383 770	55 149 794 (2.93%)
DNA element	58 022	14 563 432 (0.77%)
Small RNA	20 997	1 537 853 (0.08%)
Simple repeat	161 246	32 547 245 (1.73%)
Low complexity	326 399	21 446 303 (1.14%)
Unclassifed	1 522 272	265 603 066 (14.1%)

Genes were predicted through three different algorithms: *ab initio*, RNA-seq transcript based, and protein homology-based. For RNA-seq transcript based prediction, transcriptome data from six organ tissues (Table 4) were aligned to the assembled genome sequence using Tophat [24], and transcript structure was predicted through Cufflinks [25]. The homology-based method employs complete protein sequences from diverse taxonomical genomes, which is fit to our model. For *H. discus hannai*, the following eight species were utilized: *L. gigantea*, *Crassostrea gigas*, *A. california*, *Strongylocentrtus purpuratus*, *Branchiostoma floridae*, *Danio rerio*, *Oncorhynchus mykiss*, *and Homo sapiens*. Those protein sequences were aligned to the *H. discus hannai* genome using TBASTN (E-value ≤ 1E-4) [26]. Next, the homologous genome sequences were aligned to the matched proteins using Exonerate [27] to predict the accurate spliced alignments. Table 5 summarizes the alignment results of known proteins in various species. For *ab initio* gene prediction, Augustus [28] was trained using RNA-seq data and known proteins by using the complete transcriptome as training matrix for HMM. Fgenesh [29] and Geneid [30] were also used. The parameters used and the number of predicted genes is provided in Table 6. Gene prediction data from each method was combined using EVM (Evidence Modeler) [31] to build a consensus gene set for the abalone genome. All gene models were converted to EVM compatible GFF3 format and merged to a consensus gene set. After consensus gene annotation was generated from EVM, manual curation was conducted for abandon genes from EVM to build a final consensus gene set of *H. discus hannai*. Manual curation was performed based on the genomic DNA mapping position of the RNA-seq sequence and the protein sequence of the related species. To determine the exon-intron edge of the gene, the genome mapping information of the transcriptome sequence was firstly reflected, and if not, the mapping information of the protein sequence of the related species was referred to secondarily to confirm the gene model. Finally, genes that were not translated into protein sequences in the final gene model were removed. A total of 29 449 genes was predicted in the *H. discus hannai* genome and summary statistics for the consensus gene set is provided in Table 7. To evaluate the quality of the *H. discus hannai* draft genome, we conducted paired-end read remapping and BUSCO (Benchmarking Universal Single-Copy Orthologs) analysis. 94.89% of paired-end reads with a 350-bp insert size were successfully mapped to the assembled genome and assembled genome contains 609 complete and 130 fragmented genes in BUSCO analysis. The detailed information of BUSCO analysis is summarized in Table 8.

**Table 4.** Summary statistics of generated transcriptome data for six organ tissues using Illumina platform.

Library name	Library type	Platform	Read length	No. read	Total bp
Blood	Paired-end	Hiseq2000	101	53 525 950	5 406 120 950
Digestive duct	Paired-end	Hiseq2000	101	56 485 666	5 705 052 266
Gill	Paired-end	Hiseq2000	101	66 415 882	6 708 004 082
Hepatopancreas	Paired-end	Hiseq2000	101	58 467 176	5 905 184 776
Mantle	Paired-end	Hiseq2000	101	65 741 776	6 639 919 376
Ovary	Paired-end	Hiseq2000	101	60 997 100	6 160 707 100
Total					36 524 988 550

**Table 5.** Summary statistics of protein alignment using tBlastn for protein based evidence gene structure.

			Total	Count/	Total	Mean	Genome
Species	Type	Element	count	gene	length, bp	length, Bp	coverage, %
*Homo sapiens*	Protein	Transcript	18 792		109 068 639	5803.99	5.80
	(69 002)	Exon	77 320	4.11	12 667 395	163.83	0.67
*Danio rerio*	Protein	Transcript	11 605		68 796 463	5928.17	3.66
	(42 474)	Exon	47 300	4.08	7 978 167	168.67	0.42
*Oncorhynchus mykiss*	Protein	Transcript	15 901		55 043 032	3461.61	2.93
	(53 876)	Exon	46 040	2.90	7 567 059	164.36	0.40
*Lottia gigantea*	Protein	Transcript	29 345		177 851 531	6060.71	9.47
	(23 851)	Exon	118 165	4.03	20 583 999	174.20	1.10
*Crassostrea gigas*	Protein	Transcript	32 978		231 175 282	7009.98	12.30
	(28 027)	Exon	140 784	4.27	23 649 828	167.99	1.26
*Aplysia californica*	Protein	Transcript	10 570		67 396 621	6376.22	3.59
	(29 096)	Exon	45 737	4.33	7 797 503	170.49	0.42
*Strongylocentrotus purpuratus*	Protein	Transcript	9116		46 270 640	5075.76	2.46
	(38 730)	Exon	34 572	3.79	5 627 082	162.76	0.30
*Branchiostoma floridae*	Protein	Transcript	27 438		125 307 206	4566.92	6.67
	(58 493)	Exon	92 426	3.37	15 483 164	167.52	0.82

**Table 6.** Summary statistics for ab initio gene prediction results using various programs and parameters.

			Total	Count/	Total	Mean	Genome
Program	Matrix	Element	count	gene	length, bp	length, bp	coverage, %
Augustus	Custom parameter (RNAseq)	Gene	88 825	3.92	367 066 732	4132.47	19.54
		CDS	348 528		76 388 076	219.17	4.07
	Custom parameter (*H.discus hannai* IsoSeq)	Gene	90 396	4.11	395 511 710	4375.32	21.05
		CDS	371 487		78 508 401	211.34	4.18
	Custom parameter (H.discus discus IsoSeq)	Gene	84 322	3.97	346 455 180	4108.72	18.44
		CDS	335 103		72 527 841	216.43	3.86
	Custom parameter (BUSCO)	Gene	111 058	4.24	626 749 935	5643.45	33.36
		CDS	470 839		84 333 972	179.11	4.49
	Custom parameter (CEGAM)	Gene	76 504	4.95	393 121 657	5138.58	20.92
		CDS	378 485		63 424 677	167.58	3.38
	Custom parameter (Protein)	Gene	22 420	3.43	184 289 721	8219.88	9.81
		CDS	76 848		20 291 739	264.05	1.08
Fgenesh	Custom parameter	Gene	184 051	3.46	1 366 924 540	7426.88	72.75
		CDS	636 568		98 055 591	154.04	5.22
Geneid	*Ciona intestinalis*	Gene	789 540	1.41	436 990 370	553.47	23.26
		CDS	1 112 959		140 976 492	126.67	7.50

**Table 7.** Summary statistics for the consensus gene set of *Haliotis discus hannai* genome.

Element	No. elements	Exon/transcript	Avg. length	Total length	Genome coverage
Gene	29 449	–	2705	79 661 536	4.2%
Exon	74 745	2.54	280	20 985 298	1.1%
Intron	45 296	1.54	1295	58 676 238	3.1%

**Table 8.** Summary statistics of Benchmarking Universal Single-Copy Orthologs (BUSCO) analysis for *H. discus hannai* genome based on Metazoans DB.

Categories	#Genes	Percentage
Complete single-copy BUSCOs	609	72.2
Complete duplicate BUSCOs	48	5.7
Fragmented BUSCOs	130	15.4
Missing BUSCOs	104	12.3

In summary, here we report the first annotated Haliotidae genome of *H. discus hannai* based on various genetic evidence. We expect that the *H. discus hannai genome* presented here, which is the first genome to be sequenced in the family Haliotidae, will provide useful genomic information for many researchers. *H. discus hannai* is a cold-water abalone breed that has difficulties dealing with the change in their inhabitable latitude, which is due to global warming and the resulting increase in the rate of sudden perishing. Genomic information of abalone is essential information that can be used for genetic breeding to improve productivity and genetic engineering for the heat resistance breed. It can also provide valuable information for future genomic studies, because only limited genome information about marine animals and mollusks is currently available. Evolutionary signatures recorded in the abalone genome can be identified through future comparative genomic studies and we expect our result will provide more insight into Haliotidae and marine mollusk evolution.

## Availability of supporting data

Raw data is available in project accession PRJNA317403 in the NCBI database. Further supporting data can be found in the *GigaScience* GigaDB [32].

## List of abbreviations

EVM - Evidence Modeler

BUSCO - Benchmarking Universal Single-Copy Orthologs

## Competing interests

All authors report no competing interests.

## Author contributions

Sampling - Bo-Hye Nam, Young-Ok Kim, Dong-Gyun Kim

Sequencing - Bo-Hye Nam, Hee Jeong Kong, Woo-Jin Kim, Jeong-Ha Kang, Ji-Young Moon, Choul Ji Park, Duk Kyung Kim

Genome assembly - Bo-Hye Nam, Woori Kwak, Jae Woong Yu, Joon Yoon, SaetByeol Lee, Samsun Sung, Chul Lee, Sojeong Ka, Kelsey Caetano-Anolles

Repeat element analysis - Woori Kwak, Minseok Seo, Kwondo Kim

Gene prediction - Woori Kwak, Younhee Shin, Myunghee Jung, Byeong-Chul Kang, Ga-hee Shin

Funding and experimental design – Jung Youn Park, Cheul Min An, Seoae Cho, Heebal Kim

## Additional files

Figure S1. Tree map for sum of repeat element for *H. discus hannai*.

Figure S2. Comparison of SINE element distribution of *H. discus hannai* and *L. gigantea*.

Figure S3. Comparison of LINE element distribution of *H. discus hannai* and *L. gigantea*.

Figure S4. Comparison of LTR element distribution of *H. discus hannai* and *L. gigantea*.

Figure S5. Comparison of DNA transposon element distribution of *H. discus hannai* and *L. gigantea*.

## Supplementary Material

GIGA-D-16-00149_Original_Submission.pdf

GIGA-D-16-00149_Revision_1.pdf

Response_to_reviewer_comments_Original_Manuscript.pdf

Reviewer_1_Report_(Oiriginal_Submission).pdf

Reviewer_2_Report_(Original_Submission).pdf

Reviewer_3_Report_(Original_Submission).pdf

Supplemental material Figure S1. Tree map for sum of repeat element for *H. discus hannai*.
Figure S2. Comparison of SINE element distribution of *H. discus hannai* and *L. gigantea*.
Figure S3. Comparison of LINE element distribution of *H. discus hannai* and *L. gigantea*.
Figure S4. Comparison of LTR element distribution of *H. discus hannai* and *L. gigantea*.
Figure S5. Comparison of DNA transposon element distribution of *H. discus hannai* and *L. gigantea*.

## References

[1] Appeltans W, Bouchet P, Boxshall G World Register of Marine Species. 2012 http://www.marinespecies.org (28 February 2014, date last accessed).

[2] Hamer P, Jenkins G, Womersley B, Understanding the ecological role of abalone in the reef ecosystem of Victoria. Fish Res Rep. 2010; 132p.

[3] Elliott NG Genetic improvement programmes in abalone: what is the future? Aqua Res. 2000;31(1):51–9.

[4] Gordon HR, Cook PA World abalone fisheries and aquaculture update: supply and market dynamics. J Shellfish Res. 2004;23(4):935–40.

[5] Cook PA The worldwide abalone industry. Modern Econ. 2014;5(13):1181.

[6] Suleria HR, Masci P, Gobe G Therapeutic potential of abalone and status of bioactive molecules: a comprehensive review. Crit Rev Food Sci Nutr. 2015;57:1742–48. 10.1080/10408398.2015.1031726 26114550

[7] Lim SY Cytotoxic and antioxidant activities of abalone (Haliotis discus hannai) extracts. J Life Sci. 2014;24(7):737–42.

[8] Lee C-G, Kwon HK, Ryu JH Abalone visceral extract inhibit tumor growth and metastasis by modulating Cox-2 levels and CD8+ T cell activity. BMC Complement Alt Med. 2010;10(1):1. 10.1186/1472-6882-10-60 PMC2972231 20961430

[9] Andrews S FastQC a quality-control tool for high-throughput sequence data. 2014 http://www.bioinformatics.babraham.ac.uk/projects/fastqc/ 2 April 2015, date last access.

[10] Bolger AM, Lohse M, Usadel B Trimmomatic: a flexible trimmer for Illumina sequence data. Bioinformatics. 2014:btu170. 10.1093/bioinformatics/btu170 PMC4103590 24695404

[11] O’Connell J, Schulz-Trieglaff O, Carlson E NxTrim: optimized trimming of Illumina mate pair reads. Bioinformatics. 2015 31(12):2035–37. 25661542 10.1093/bioinformatics/btv057

[12] Marçais G, Kingsford C A fast, lock-free approach for efficient parallel counting of occurrences of k-mers. Bioinformatics. 2011;27(6):764–70. 21217122 10.1093/bioinformatics/btr011 PMC3051319

[13] Boetzer M, Henkel CV, Jansen HJ Scaffolding pre-assembled contigs using SSPACE. Bioinformatics. 2011;27(4):578–79. 21149342 10.1093/bioinformatics/btq683

[14] English AC, Richards S, Han Y Mind the gap: upgrading genomes with Pacific Biosciences RS long-read sequencing technology. PloS One. 2012;7(11):e47768. 23185243 10.1371/journal.pone.0047768 PMC3504050

[15] Luo R, Liu B, Xie Y SOAPdenovo2: an empirically improved memory-efficient short-read de novo assembler. GigaScience. 2012;1(1):1–6. 23587118 10.1186/2047-217X-1-18 PMC3626529

[16] Tarailo‐ Graovac M, Chen N Using RepeatMasker to identify repetitive elements in genomic sequences. Curr Protoc Bioinformatics. 2009:4.10. 1–4.10. 14. 10.1002/0471250953.bi0410s25 19274634

[17] Jurka J, Kapitonov VV, Pavlicek A Repbase Update, a database of eukaryotic repetitive elements. Cyt Genome Res. 2005;110(1–4):462–67. 10.1159/000084979 16093699

[18] Bao Z, Eddy SR Automated de novo identification of repeat sequence families in sequenced genomes. Genome Res. 2002;12(8):1269–76. 12176934 10.1101/gr.88502 PMC186642

[19] Price AL, Jones NC, Pevzner PA De novo identification of repeat families in large genomes. Bioinformatics. 2005;21(suppl 1):i351–i358. 15961478 10.1093/bioinformatics/bti1018

[20] Benson G Tandem repeats finder: a program to analyze DNA sequences. Nucleic Acids Res. 1999;27(2):573. 9862982 10.1093/nar/27.2.573 PMC148217

[21] Bailly-Bechet M, Haudry A, Lerat E “One code to find them all”: a perl tool to conveniently parse RepeatMasker output files. Mobile DNA. 2014; 5(1):1.24382139

[22] Chénais B, Caruso A, Hiard S The impact of transposable elements on eukaryotic genomes: from genome size increase to genetic adaptation to stressful environments. Gene. 2012;509(1):7–15. 22921893 10.1016/j.gene.2012.07.042

[23] Kidwell MG Transposable elements and the evolution of genome size in eukaryotes. Genetica. 2002;115(1):49–63. 12188048 10.1023/a:1016072014259

[24] Trapnell C, Pachter L, Salzberg SL TopHat: discovering splice junctions with RNA-Seq. Bioinformatics. 2009;25(9):1105–11. 19289445 10.1093/bioinformatics/btp120 PMC2672628

[25] Trapnell C, Roberts A, Goff L Differential gene and transcript expression analysis of RNA-seq experiments with TopHat and Cufflinks. Nat Protocols. 2012;7(3):562–78. 22383036 10.1038/nprot.2012.016 PMC3334321

[26] Altschul SF, Madden TL, Schäffer AA Gapped BLAST and PSI-BLAST: a new generation of protein database search programs. Nucleic Acids Res. 1997;25(17):3389–402. 9254694 10.1093/nar/25.17.3389 PMC146917

[27] Slater GS, Birney E Automated generation of heuristics for biological sequence comparison. BMC Bioinformatics. 2005;6(1):31. 15713233 10.1186/1471-2105-6-31 PMC553969

[28] Stanke M, Diekhans M, Baertsch R Using native and syntenically mapped cDNA alignments to improve de novo gene finding. Bioinformatics. 2008;24(5):637–44. 18218656 10.1093/bioinformatics/btn013

[29] Solovyev V, Kosarev P, Seledsov I *Automatic annotation of eukaryotic genes, pseudogenes and promoters* . Genome Biol. 2006;7(Suppl 1):S10. 16925832 10.1186/gb-2006-7-s1-s10 PMC1810547

[30] Blanco E, Parra G, Guigó R Using geneid to identify genes. Curr Protoc Bioinformatics. 2007;4.3. 1–4.3. 28. 10.1002/0471250953.bi0403s18 18428791

[31] Haas BJ, Salzberg SL, Zhu W Automated eukaryotic gene structure annotation using EVidenceModeler and the Program to Assemble Spliced Alignments. Genome Biol. 2008;9(1):R7. 18190707 10.1186/gb-2008-9-1-r7 PMC2395244

[32] Nam B, Kwak W, Kim Y Supporting data for “Genome sequence of pacific abalone (Haliotis discus hannai): the first draft genome in family Haliotidae” GigaScience Database. 2017 http://dx.doi.org/10.5524/100281. 10.1093/gigascience/gix014 PMC5439488 28327967

