# Supplementary material for: Genome sequence of pacific abalone (Haliotis discus hannai): the first draft genome in family Haliotidae
Source: Gigascience. 2017 Mar 7;6(5):1–8. doi: 10.1093/gigascience/gix014 (PMC5439488; doi:10.1093/gigascience/gix014)
Supplement: GIGA-D-16-00149_Original_Submission.pdf [file gix014_GIGA-D-16-00149_Original_Submission.pdf]

Manuscript

Click here to download Manuscript Manuscript_FIN2_ENG.docx

Genome sequence of pacific abalone (*Haliotis discus hannai*): the first draft genome in family Haliotidae.

Bo-Hye Nam[1†], Woori Kwak[2,3†], Young-Ok Kim[1], Dong-Gyun Kim[1], Hee Jeong Kong[1], Woo-Jin Kim[1], Jeong-Ha Kang[1], Jung Youn Park[1], Cheul Min An[1], Ji-Young Moon[1], Choul Ji Park[4], Jae Woong Yu[3], Joon Yoon[2], Minseok Seo[3], Kwondo Kim[2,3], Duk Kyung Kim[3], SaetByeol Lee[3], Samsun Sung[3], Chul Lee[2,3], Younhee Shin[5], Myunghee Jung[5], Byeong-Chul Kang[5], Ga-hee Shin[5], Sojeong Ka[6], Kelsey Caetano-Anolles[6], Seoae Cho[3*] and Heebal Kim[7*]

[1]Biotechnology Research Division, National Institute of Fisheries Science, Haean-ro 216, Gijang-eup, Gijang-gun, Busan 619-705, Korea; [2]Interdisciplinary Program in Bioinformatics, Seoul National University, Seoul 151-747, Republic of Korea; [3]C&K Genomics, Main Bldg. #514, SNU Research Park, Seoul 151-919, Republic of Korea; [4]Genetics and Breeding Research Center, National Institute of Fisheries Science, Geoje, Gyeongsangnam-do 656-842, Republic of Korea; [5]Research and Development Center, Insilicogen Inc., Yongin-si 16954, Gyeonggi-do, Republic of Korea; [6]Animal Science and Biotechnology, Seoul National University, Seoul 151-747, Republic of Korea; [7]Department of Agricultural Biotechnology and Research Institute for Agriculture and Life Sciences, Seoul National University, Seoul 151-921, Republic of Korea

Bo-Hye Nam: nambohye@korea.kr; Woori Kwak : asleo@cnkgenomics.com; Young-Ok Kim : yobest12@korea.kr; Dong-Gyun Kim : combikola@korea.kr; Hee Jeong Kong : heejkong@korea.kr; Woo-Jin Kim : wj2464@korea.kr; Jeong-Ha Kang : genetics@korea.kr;

Jung Youn Park : genome@korea.kr; Cheul Min An : ancm@korea.kr; Ji-Young Moon: moonjy@nfrdi.go.kr; Cheol-Ji Park : choulji@kore.kr; Jae Woong Yu : jwyu@cnkgenomics.com; Joon Yoon : joonyoon.jay@gmail.com; Minseok Seo : nijorral@gmail.com; Kwondo Kim : bigkd@snu.ac.kr; Duk Kyung Kim : duk-kyung@cnkgenomics.com; SaetByeol Lee : sblee@cnkgenomics.com; Samsun Sung : triples@cnkgenomics.com; Chul Lee : swear0712@naver.com; Younhee Shin : yhshin@insilicogen.com; Myunghee Jung : mhjung@insilicogen.com; Byeong-Chul Kang : bckang@insilicogen.com; Ga-hee Shin : ghshin@insilicogen.com; Sojeong Ka : skasnu@snu.ac.kr; Kelsey Caetano-Anolles : kelseyca@gmail.com; Seoae Cho : seoae@cnkgenomics.com; Heebal Kim : heebal@snu.ac.kr

† These authors equally contributed and should be regarded as co-first authors.

* Corresponding authors

Seoae Cho

C&K Genomics

Main Bldg. #423, SNU Research Park,

Seoul 151-919, Republic of Korea

Phone : +82-2-876-8820

Fax : +82-2-876-8827

E-mail: seoae@cnkgenomics.com

Heebal Kim

Research Institute of Agriculture and Life Sciences

Seoul National University, San 56-1, Sillim-dong, Gwanak-gu, Seoul 151-742, Korea

Phone : +82-2-880-4803

Fax : +82-2-883-8812

E-mail: heebal@snu.ac.kr

Abstract

Background

Abalones are large marine snails in the family Haliotidae and the genus Haliotis belonging to the class Gastropoda of the phylum Mollusca. The family Haliotidae contains only one genus, Haliotis, and this single genus is known to contain several species of abalone. With 18 additional subspecies, the most comprehensive treatment of Haliotidae considers 56 species valid[1]. Abalone is an economically important fishery and aquaculture animal which is considered a highly-prized seafood delicacy. The total global supply of abalone has increased fivefold since 1970's. In order to prevent indiscreetly fishing abalones, legal landings from abalone fisheries have made fishery productions decreased gradually from 19,720 mt to 7,486 mt, but have made farm productions increase explosively from 50 mt to 103,464 mt in the past forty years [2]. Additionally, researchers have recently focused on Abalone given their reported tumor suppression effect [3-5]. However, despite the valuable features of this marine animal, no genomic information is available for Haliotidae family and related research is still limited.

Findings

In order to construct the *H.discus hannai* genome, a total of 580G base pairs using Illumina and Pacbio platforms were generated with 322-fold coverage based on the 1.8Gb estimated genome size of *H.discus hannai* using flow cytometry. The final genome assembly consisted of 1.86Gb with 35,450 scaffolds (>2kb). GC content level was 40.51%, and the N50 length of assembled scaffolds was 211kb. We identified 29,449 genes using Evidence Modeler based on the gene information from ab initio prediction, protein homology with known genes and transcriptome evidence of RNA-seq.

Conclusions

Here we present the first Haliotidae genome, *Haliotis discus hannai*, with sequencing data, assembly, and gene annotation information. This will be helpful for resolving the lack of genomic information in the Haliotidae family as well as providing more opportunities for understanding gastropod evolution.

Keywords

Abalone genome, Halotidae, *Haliotis discus hannai*

Data description

Abalone is one of the most important marine gastropod molluscs that inhabits various coastal regions of the world. It is well known that abalone habitation impacts algal communications connected with the reef ecosystem, so they are often utilized for ecological research[6]. Among many abalone species, *H.discus hannai* is a widely used ingredient in East Asian cuisine. It is a valuable food resource due to its richness in protein and other nutrients[7, 8] and it is considered as an important fishery and aquaculture animal. Additionally, researchers have recently focused on *H.discus hannai* given their reported tumor suppression effect [3-5] However, despite the valuable features of this marine animal, no genomic information is available. Therefore, the first draft genome in family Haliotidae has the potential to be utilized as a valuable resource for many researchers.

A single *Haliotis discus hannai* abalone was collected from the brood stock at the Genetic and Breeding Research Center (GBRC) of the National Fisheries Research & Development Institute (NFRDI) on Geoje Island, Korea for sampling. Hemolymph(10ml) was withdrawn from the sole side foot muscle using a syringe. For genomic DNA extraction, hemocytes were harvested from fresh hemolymph by centrifugation at 3000 × rpm for 5 min at 4℃. Genomic DNA was extracted using a DNeasy Animal Mini Kit (Qiagen, Hilden, Germany). A total 39.38 ug of DNA was quantified using the standard procedure of Quant-iT PicoGreen dsDNA Assay Kit (Molecular Probes, Eugene, OR, USA) with Synergy HTX Multi-Mode Reader (Biotek, Winooski, VT, USA). Quality of DNA was also checked using ND-1000 spectrophotometer (Thermo Scientifc, Wilmington, DE, USA).

For whole genome shotgun sequencing and draft genome assembly, we used multiple sequencing platforms (Illumina Hiseq2000, Nextseq500 and Pacbio RS II) with 7 different

libraries. First, two paired-end libraries with insert sizes of 250bp and 350bp were constructed using Illumina TruSeq DNA Sample Prep. Kit (Illumina, San Diego, CA). Mate pair libraries with insert sizes of about 3k, 5k, 8k, and 10k were constructed for scaffolding process using Illumina Nextera mate-pair library construction protocol (Illumina, San Diego, CA). For high-quality genome assembly, long mate pair library with insert size over 40kb is essential. We tried to construct a long mate pair library using 40kb fosmid clone. However, efficiency of fosmid library construction was very low and we could not retain enough amount of clone. Therefore, Pacbio system was employed for final scaffolding process using long read. Pacbio long reads were generated using P6-C4 chemistry of Pacbio RS II system. Detailed information about the constructed library and generated sequencing data is provided in Table 1. Quality control process of generated raw data was conducted for downstream analysis. Quality of raw data was checked using FASTQC[9] and adapter sequences were removed via Trimmomatic[10], for paired-end libraries, and Nxtrim[11], for mate-pair libraries. K-mer frequency analysis of the abalone genome was conducted using a paired-end library with 350bp insert-size and the jellyfish[12] command-line program. The K-mer distribution of the paired-end library provides valuable information about the target genome. As a result, 19-mer distribution of *Haliotis discus hannai* genome was generated (Figure 1). Genome size estimation based on the 19-mer distribution was conducted through "Estimate genome size.pl" code (https://github.com/josephryan/estimate_genome_size.pl/wiki/Estimate-genome-size.pl"). The estimated genome size of *H.discus hannai* using 19-mer distribution was about 1.65Gb. Based on the 19-mer distribution of paired-end reads, there was a second peak located in the half x-axis of the main peak. This result indicates that *H.discus hannai* genome had high heterozygous genetic character and probable DNA contamination from other organisms. Before genome assembly, raw reads from Hiseq2000, Nextseq500 paired-end and mate pairs

were pre-processed by bacterial sequences, duplicates, and ambiguous nucleotides. The resulting high-quality sequences were used in subsequent assembly. Error correction and initial contig assembly was conducted using clc_assembler within the CLC Assembly Cell (http://www.clcbio.com/products/clc-assembly-cell/) software pipeline. Scaffolds were then built using the mate-pairs and Pacbio RS II reads sequentially by SSPACE[13] and PBJelly2[14]. After scaffolding, Gapcloser[15] was employed f to close the gaps within each scaffold with -l 155 and -p 31, iteratively. Summary statistics for final assembly is provided in Table 2.

Before conducting gene prediction using the assembled sequence, repeat elements were identified using RepeatMasker[16] with Repbase[17]. RepeatModeler, which includes RECON[18], RepeatScout[19] and TRF[20], was used to create a custom database of *H.discus hannai*. After custom library construction, RepeatMasker with RMBlast was used for each genome with 'no_is' option, using repeat libraries from RepeatModeler and Repbase. Identified mobile elements are summarized in Table 3.

Genes were predicted through three different algorithms: *ab initio*, RNA-seq transcript based, and protein homology-based. For RNA-seq transcript based prediction, transcriptome data from six organ tissues (Table 4) were aligned to the assembled genome sequence using Tophat[21], and transcript structure was predicted through Cufflinks[22]. The homology-based method employs complete protein sequences from diverse taxonomical genomes, which is fit to our model. For *Haliotis discus hannai*, the following 8 species were utilized: *Lottia gigantea, Crssostrea gigas, Aplysia california, Strongylocentrtus purpuratus, Branchiostoma floridae, Danio rerio, Oncorhynchus mykiss and Homo sapiens*. Those protein sequences were aligned to the *Haliotis discus hannai* genome using TBASTN (E-value ≤ 1E-4)[23]. Next, the homologous genome sequences were aligned to the matched

proteins using Exonerate[24] to predict the accurate spliced alignments. Table 5 summarizes the alignment results of known proteins in various species. For *ab initio* gene prediction, Augustus[25] was trained using RNA-seq data and known proteins by using the complete transcriptome as training matrix for HMM. Fgenesh[26] and Geneid[27] were also used. The parameters used and the number of predicted genes is provided in Table 6. Gene prediction data from each method was combined using EVM (Evidence Modeler)[28] to build a consensus gene set for the abalone genome. All gene models were converted to EVM compatible GFF3 format and merged to a consensus gene set. After consensus gene annotation was generated from EVM, manual curation was conducted for abandon genes from EVM to build a final consensus gene set of *H.discus hannai*. A total of 29,449 genes were predicted in the *H.discus hannai* genome and summary statistics for the consensus gene set is provided in Table 7. To evaluate the quality of *H.discus hannai* draft genome, we conducted paired-end read remapping and BUSCO (Benchmarking Universal Single-Copy Orthologs) analysis. 94.89% of paired-end reads with 350bp insert size were successfully mapped to the assembled genome and assembled genome contains 609 complete and 130 fragmented genes in BUSCO analysis. The detailed information of BUSCO analysis is summarized in Table 8.

In summary, here we report the first annotated Haliotidae genome of *H.discus hannai* based on various genetic evidence. We expect that the_*H.discus hannai genome* presented here, which is the first genome to be sequenced in the family Haliotidae, will provide useful genomic information for many researchers. *Haliotis discus hannai* is a cold-water abalone breed that have difficulties dealing with the change in their inhabitable latitude, which is due to global warming and the resulting increase in the rate of sudden perishing. Genomic

information of abalone is essential information which can be used for genetic breeding to improve productivity and genetic engineering for the heat resistance breed. It can also provide valuable information for future genomic studies because only limited genome information about marine animals and mollusks is currently available. Abalone has the biggest genome size among known gastropod with various and specific phenotype features. Evolutionary signatures recorded in abalone genome can be identified through future comparative genomic studies and we expect our result will provide more insight into Haliotidae and marine mollusk evolution.

Availability of supporting data

Raw data is available in project accession PRJNA317403 of NCBI database.

List of abbreviations

GBRC - Genetic and Breeding Research Center

NFRDI - National Fisheries Research & Development Institute

EVM - Evidence Modeler

BUSCO - Benchmarking Universal Single-Copy Orthologs

Competing interests

All authors report no competing interests.

Authors contributions

Sampling - Bo-Hye Nam, Young-Ok Kim, Dong-Gyun Kim

Sequencing - Bo-Hye Nam, Hee Jeong Kong, Woo-Jin Kim, Jeong-Ha Kang, Ji-Young Moon, Choul Ji Park, Duk Kyung Kim

Genome assembly - Bo-Hye Nam, Woori Kwak, Jae Woong Yu, Joon Yoon, SaetByeol Lee, Samsun Sung, Chul Lee, Sojeong Ka, Kelsey Caetano-Anolles

Repeat element analysis - Woori Kwak, Minseok Seo, Kwondo Kim

Gene prediction - Woori Kwak, Younhee Shin, Myunghee Jung, Byeong-Chul Kang, Ga-hee Shin

Funding and experimental design – Jung Youn Park, Cheul Min An, Seoae Cho, Heebal Kim

Acknowledgements

This work was supported by a grant from the National Institute of Fisheries Science (R2016024).

References

1. Appeltans, W., et al., *World register of marine species.* Accessed online: http://www. marinespecies. org (accessed on 28 February 2014), 2012.
2. Cook, P.A., *The worldwide abalone industry.* Modern Economy, 2014. **5**(13): p. 1181.
3. Lim, S.Y., *Cytotoxic and Antioxidant Activities of Abalone (Haliotis discus hannai) Extracts.* Journal of Life Science, 2014. **24**(7): p. 737-742.
4. Suleria, H.R., et al., *Therapeutic Potential of Abalone and Status of Bioactive Molecules: A Comprehensive Review.* Critical reviews in food science and nutrition, 2015(just-accepted): p. 00-00.
5. Lee, C.-G., et al., *Abalone visceral extract inhibit tumor growth and metastasis by modulating Cox-2 levels and CD8+ T cell activity.* BMC complementary and alternative medicine, 2010. **10**(1): p. 1.
6. Hamer, P.A. and F. Victoria, *Understanding the ecological role of abalone in the reef ecosystem of Victoria*. 2010: Fisheries Victoria.
7. Elliott, N.G., *Genetic improvement programmes in abalone: what is the future?* Aquaculture Research, 2000. **31**(1): p. 51-59.
8. Gordon, H.R. and P.A. Cook, *World abalone fisheries and aquaculture update: supply and market dynamics.* Journal of shellfish Research, 2004. **23**(4): p. 935-940.
9. Andrews, S., *FastQC: A quality control tool for high throughput sequence data.* Reference Source, 2010.
10. Bolger, A.M., M. Lohse, and B. Usadel, *Trimmomatic: a flexible trimmer for Illumina sequence data.* Bioinformatics, 2014: p. btu170.
11. O'Connell, J., et al., *NxTrim: optimized trimming of Illumina mate pair reads.* Bioinformatics, 2015. **31**(12): p. 2035-2037.
12. Marçais, G. and C. Kingsford, *A fast, lock-free approach for efficient parallel counting of occurrences of k-mers.* Bioinformatics, 2011. **27**(6): p. 764-770.
13. Boetzer, M., et al., *Scaffolding pre-assembled contigs using SSPACE.* Bioinformatics, 2011. **27**(4): p. 578-579.
14. English, A.C., et al., *Mind the gap: upgrading genomes with Pacific Biosciences RS long-read sequencing technology.* PloS one, 2012. **7**(11): p. e47768.
15. Luo, R., et al., *SOAPdenovo2: an empirically improved memory-efficient short-read de novo assembler.* GigaScience, 2012. **1**(1): p. 1-6.
16. Tarailo-Graovac, M. and N. Chen, *Using RepeatMasker to identify repetitive elements in genomic sequences.* Current Protocols in Bioinformatics, 2009: p. 4.10. 1-4.10. 14.
17. Jurka, J., et al., *Repbase Update, a database of eukaryotic repetitive elements.* Cytogenetic and genome research, 2005. **110**(1-4): p. 462-467.
18. Bao, Z. and S.R. Eddy, *Automated de novo identification of repeat sequence families in sequenced genomes.* Genome Research, 2002. **12**(8): p. 1269-1276.

19. Price, A.L., N.C. Jones, and P.A. Pevzner, *De novo identification of repeat families in large genomes.* Bioinformatics, 2005. **21**(suppl 1): p. i351-i358.

20. Benson, G., *Tandem repeats finder: a program to analyze DNA sequences.* Nucleic acids research, 1999. **27**(2): p. 573.

21. Trapnell, C., L. Pachter, and S.L. Salzberg, *TopHat: discovering splice junctions with RNA-Seq.* Bioinformatics, 2009. **25**(9): p. 1105-1111.

22. Trapnell, C., et al., *Differential gene and transcript expression analysis of RNA-seq experiments with TopHat and Cufflinks.* Nature protocols, 2012. **7**(3): p. 562-578.

23. Altschul, S.F., et al., *Gapped BLAST and PSI-BLAST: a new generation of protein database search programs.* Nucleic acids research, 1997. **25**(17): p. 3389-3402.

24. Slater, G.S. and E. Birney, *Automated generation of heuristics for biological sequence comparison.* BMC bioinformatics, 2005. **6**(1): p. 31.

25. Stanke, M., et al., *Using native and syntenically mapped cDNA alignments to improve de novo gene finding.* Bioinformatics, 2008. **24**(5): p. 637-644.

26. Solovyev, V., et al., *Automatic annotation of eukaryotic genes, pseudogenes and promoters.* Genome Biol, 2006. **7**(Suppl 1): p. S10.

27. Blanco, E., G. Parra, and R. Guigó, *Using geneid to identify genes.* Current protocols in bioinformatics, 2007: p. 4.3. 1-4.3. 28.

28. Haas, B.J., et al., *Automated eukaryotic gene structure annotation using EVidenceModeler and the Program to Assemble Spliced Alignments.* Genome biology, 2008. **9**(1): p. R7.

Tables

Table 1. Summary statistics of generated whole genome shotgun sequencing data.

Library Name	Library Type	Insert Size	Platform	Read Length	No. Read	Total bp
250bp	Paired-end	250	Nextseq500	150	876,529,480	131,440,418,087
350bp	Paired-end	350	Hiseq2000	101	1,413,620,786	142,775,699,386
3k	Mate-pair	3,000	Nextseq500	150	580,064,464	85,689,154,056
5k	Mate-pair	5,000	Nextseq500	150	468,432,888	69,966,139,205
8k	Mate-pair	8,000	Nextseq500	150	335,132,792	50,109,845,012
10k	Mate-pair	10,000	Nextseq500	150	569,376,096	85,080,237,236
20k	P6-C4	20,000	Pacbio RS II	10,094 (average)	1,573,020	15,879,626,978
**Total**						580,941,119,960

Table 2. Summary statistics for the *Haliotis discus hannai* draft genome (>2kb).

**Assembled Genome**	
Size(1n)	1.80 Gb
GC level	40.51%
No. scaffolds	35,450
N50 of scaffolds (bp)	211,346
N bases in scaffolds (%)	116 Mb (6.45%)
Longest(shortest) scaffolds (bp)	2,207,537 (2,000)
Average scaffold Length (bp)	50,870.65

**Repeat Element**	No. Element	Length (%)
SINE	284,485	96,155,199 (5.11%)
LINE	700,245	160,387,248 (8.53%)
LTR element	383,770	55,149,794 (2.93%)
DNA element	58,022	14,563,432 (0.77%)
Small RNA	20,997	1,537,853 (0.08%)
Simple repeat	161,246	32,547,245 (1.73%)
Low complexity	326,399	21,446,303 (1.14%)
Unclassifed	1,522,272	265,603,066 (14.1%)

Table 3. Summary of identified repeat elements in the *Haliotis discus hannai* genome.

Table 4. Summary statistics of generated transcriptome data for six organ tissues using Illumina platform.

Library Name	Library Type	Platform	Read Length	No. Read	Total bp
Blood	Paired-end	Hiseq2000	101	53,525,950	5,406,120,950
Digestive duct	Paired-end	Hiseq2000	101	56,485,666	5,705,052,266
Gill	Paired-end	Hiseq2000	101	66,415,882	6,708,004,082
Hepatopancreas	Paired-end	Hiseq2000	101	58,467,176	5,905,184,776
Mantle	Paired-end	Hiseq2000	101	65,741,776	6,639,919,376
Ovary	Paired-end	Hiseq2000	101	60,997,100	6,160,707,100
Total					36,524,988,550

Species	Type	Element	Total count	Count/ Gene	Total length, bp	Mean length, Bp	Genome Coverage %

Table 5. Summary statistics of protein alignment using tBlastn for protein based evidence gene structure.

Homo sapiens	Protein (69,002)	Transcript	18,792		109,068,639	5,803.99	5.80
		Exon	77,320	4.11	12,667,395	163.83	0.67
Danio rerio	Protein (42,474)	Transcript	11,605		68,796,463	5,928.17	3.66
		Exon	47,300	4.08	7,978,167	168.67	0.42
Oncorhynchus mykiss	Protein (53,876)	Transcript	15,901		55,043,032	3,461.61	2.93
		Exon	46,040	2.90	7,567,059	164.36	0.40
Lottia gigantea	Protein (23,851)	Transcript	29,345		177,851,531	6,060.71	9.47
		Exon	118,165	4.03	20,583,999	174.20	1.10
Croatea gigas	Protein (28,027)	Transcript	32,978		231,175,282	7,009.98	12.30
		Exon	140,784	4.27	23,649,828	167.99	1.26
Aplysia californica	Protein (29,096)	Transcript	10,570		67,396,621	6,376.22	3.59
		Exon	45,737	4.33	7,797,503	170.49	0.42
Strongylocentrotus purpuratus	Protein (38,730)	Transcript	9,116		46,270,640	5,075.76	2.46
		Exon	34,572	3.79	5,627,082	162.76	0.30
Branchiostoma floridae	Protein (58,493)	Transcript	27,438		125,307,206	4,566.92	6.67
		Exon	92,426	3.37	15,483,164	167.52	0.82

Table 6. Summary statistics for ab initio gene prediction results using various programs and parameters.

Program	Matrix	Element	Total count	Count/Gene	Total length, bp	Mean length, bp	Genome Coverage %
Augustus	Custom parameter (RNAseq)	Gene	88,825	3.92	367,066,732	4,132.47	19.54
		CDS	348,528		76,388,076	219.17	4.07
	Custom parameter (*H.discus hannai* IsoSeq)	Gene	90,396	4.11	395,511,710	4,375.32	21.05
		CDS	371,487		78,508,401	211.34	4.18
	Custom parameter (H.discus discus IsoSeq)	Gene	84,322	3.97	346,455,180	4,108.72	18.44
		CDS	335,103		72,527,841	216.43	3.86
	Custom parameter (BUSCO)	Gene	111,058	4.24	626,749,935	5,643.45	33.36
		CDS	470,839		84,333,972	179.11	4.49
	Custom parameter (CEGAM)	Gene	76,504	4.95	393,121,657	5,138.58	20.92
		CDS	378,485		63,424,677	167.58	3.38
	Custom parameter (Protein)	Gene	22,420	3.43	184,289,721	8,219.88	9.81
		CDS	76,848		20,291,739	264.05	1.08
Fgenesh	Custom parameter	Gene	184,051	3.46	1,366,924,540	7,426.88	72.75
		CDS	636,568		98,055,591	154.04	5.22
Geneid	*Ciona intestinalis*	Gene	789,540	1.41	436,990,370	553.47	23.26
		CDS	1,112,959		140,976,492	126.67	7.50

Table 7. Summary statistics for the consensus gene set of *Haliotis discus hannai* genome.

Element	No. elements	Exon/transcript	Avg. length	Total length	Genome coverage

Gene	29,449	-	2,705	79,661,536	4.2 %
Exon	74,745	2.54	280	20,985,298	1.1 %
Intron	45,296	1.54	1,295	58,676,238	3.1 %

Table 8. Summary statistics of Benchmarking Universal Single-Copy Orthologs (BUSCO) analysis for *Haliotis discus hannai* genome based on Metazoans DB.

Categories	#Genes	Percentage
Complete Single-Copy BUSCOs	609	72.2%
Complete Duplicate BUSCOs	48	5.7%
Fragmented BUSCOs	130	15.4%

Missing BUSCOs	104	12.3%

Figures

Figure 1. 19-mer distribution of using jellyfish with 350bp paired-end whole genome sequencing data.

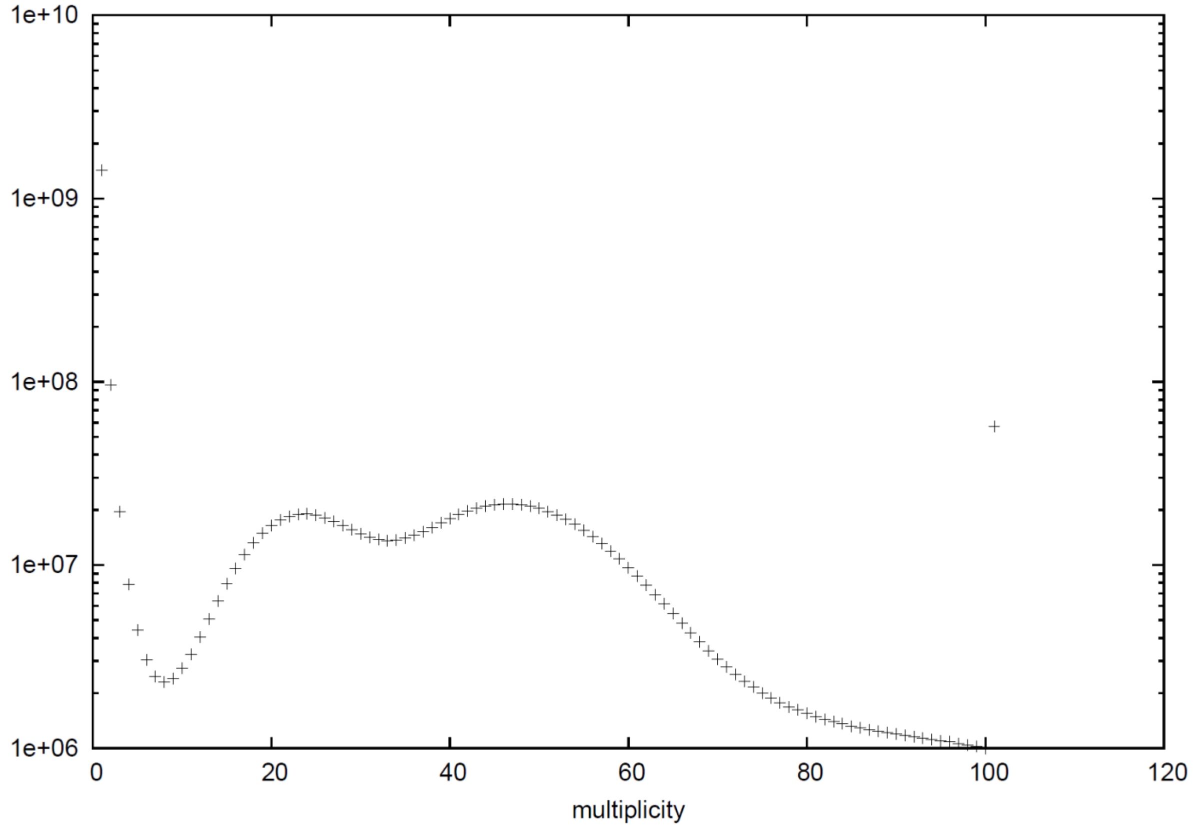

Personal Cover Click here to download Personal Cover Cover_letter.docx

SEOUL NATIONAL UNIVERSITY

Laboratory of Bioinformatics and Population Genetics

Heebal Kim, Ph.D.
Professor

November, 21, 2016

Editor in Chief, *GIGA SCIENCE*

Dear. Editor

We are pleased to submit our manuscript entitled “Genome sequence of pacific abalone (Haliotis discus hannai): the first draft genome in family Haliotidae.” by Nam et al, for publication as a data note in *GIGA SCIENCE*.

While many genome studies were successfully conducted for various species, no genomics information is available for abalone which is evolutionary and ecologically important marine animal. To solve the limitation of previous studies, we conducted the large-scale genome analysis for the abalone and constructed draft genome with annotation. This is the first Haliotidae genome ever analyzed and this will be useful resources for related researchers. In addition, we expect our data to be a valuable resource for comparative genomic studies because only few genomic information is available for marine mollusks in current genome project process. Therefore, we hope that editor in chief positively consider our manuscript.

The manuscript attached has been seen and approved by all author contributors. Feel free to get in touch with me if you have any questions.

Yours sincerely,

Heebal Kim on behalf of all the authors

Laboratory of Bioinformatics and Population Genetics, Department of Agricultural Biotechnology, Seoul National University, San 56-1, Sillim-dong, Gwanak-gu, Seoul 151-742, Korea Phone: 82-2-880-4803, Fax: 82-2-883-8812, Email: heebal@snu.ac.kr

Dept of Agricultural Biotechnology
Seoul National University
San 56-1 Daehak-dong, Gwanak-gu, Seoul 151-742
KOREA

T +82 2 880 4803
F +82 2 883 8812
heebal@snu.ac.kr
