# Supplementary material for: Genome sequence of pacific abalone (Haliotis discus hannai): the first draft genome in family Haliotidae
Source: Gigascience. 2017 Mar 7;6(5):1–8. doi: 10.1093/gigascience/gix014 (PMC5439488; doi:10.1093/gigascience/gix014)
Supplement: GIGA-D-16-00149_Revision_1.pdf [file gix014_GIGA-D-16-00149_Revision_1.pdf]

Manuscript Click here to download Manuscript Revised_FIN with DOI.docx

# Genome sequence of pacific abalone (*Haliotis discus hannai*): the first draft genome in family Haliotidae.

**Bo-Hye Nam[1†], Woori Kwak[2,3†], Young-Ok Kim[1], Dong-Gyun Kim[1], Hee Jeong Kong[1], Woo-Jin Kim[1], Jeong-Ha Kang[1], Jung Youn Park[1], Cheul Min An[1], Ji-Young Moon[1], Choul Ji Park[4], Jae Woong Yu[3], Joon Yoon[2], Minseok Seo[3], Kwondo Kim[2,3], Duk Kyung Kim[3], SaetByeol Lee[3], Samsun Sung[3], Chul Lee[2,3], Younhee Shin[5], Myunghee Jung[5], Byeong-Chul Kang[5], Ga-hee Shin[5], Sojeong Ka[6], Kelsey Caetano-Anolles[6], Seoae Cho[3*] and Heebal Kim[7*]**

[1]Biotechnology Research Division, National Institute of Fisheries Science, Haean-ro 216, Gijang-eup, Gijang-gun, Busan 619-705, Korea; [2]Interdisciplinary Program in Bioinformatics, Seoul National University, Seoul 151-747, Republic of Korea; [3]C&K Genomics, Main Bldg. #514, SNU Research Park, Seoul 151-919, Republic of Korea; [4]Genetics and Breeding Research Center, National Institute of Fisheries Science, Geoje, Gyeongsangnam-do 656-842, Republic of Korea; [5]Research and Development Center, Insilicogen Inc., Yongin-si 16954, Gyeonggi-do, Republic of Korea; [6]Animal Science and Biotechnology, Seoul National University, Seoul 151-747, Republic of Korea; [7]Department of Agricultural Biotechnology and Research Institute for Agriculture and Life Sciences, Seoul National University, Seoul 151-921, Republic of Korea

Bo-Hye Nam: nambohye@korea.kr; Woori Kwak : asleo@cnkgenomics.com; Young-Ok Kim : yobest12@korea.kr; Dong-Gyun Kim : combikola@korea.kr; Hee Jeong Kong : heejkong@korea.kr; Woo-Jin Kim : wj2464@korea.kr; Jeong-Ha Kang : genetics@korea.kr;

Jung Youn Park : genome@korea.kr; Cheul Min An : ancm@korea.kr; Ji-Young Moon: moonjy@nfrdi.go.kr; Cheol-Ji Park : choulji@kore.kr; Jae Woong Yu : jwyu@cnkgenomics.com; Joon Yoon : joonyoon.jay@gmail.com; Minseok Seo : nijorral@gmail.com; Kwondo Kim : bigkd@snu.ac.kr; Duk Kyung Kim : duk-kyung@cnkgenomics.com; SaetByeol Lee : sblee@cnkgenomics.com; Samsun Sung : triples@cnkgenomics.com; Chul Lee : swear0712@naver.com; Younhee Shin : yhshin@insilicogen.com; Myunghee Jung : mhjung@insilicogen.com; Byeong-Chul Kang : bckang@insilicogen.com; Ga-hee Shin : ghshin@insilicogen.com; Sojeong Ka : skasnu@snu.ac.kr; Kelsey Caetano-Anolles : kelseyca@gmail.com; Seoae Cho : seoae@cnkgenomics.com; Heebal Kim : heebal@snu.ac.kr

† These authors equally contributed and should be regarded as co-first authors.

* Corresponding authors

Seoae Cho

C&K Genomics

Main Bldg. #423, SNU Research Park,

Seoul 151-919, Republic of Korea

Phone : +82-2-876-8820

Fax : +82-2-876-8827

E-mail: seoae@cnkgenomics.com

Heebal Kim

Research Institute of Agriculture and Life Sciences

Seoul National University, San 56-1, Sillim-dong, Gwanak-gu, Seoul 151-742, Korea

Phone : +82-2-880-4803

Fax : +82-2-883-8812

E-mail: heebal@snu.ac.kr

# Abstract

## Background

Abalones are large marine snails in the family Haliotidae and the genus Haliotis belonging to the class Gastropoda of the phylum Mollusca. The family Haliotidae contains only one genus, Haliotis, and this single genus is known to contain several species of abalone. With 18 additional subspecies, the most comprehensive treatment of Haliotidae considers 56 species valid[1]. Abalone is an economically important fishery and aquaculture animal which is considered a highly-prized seafood delicacy. The total global supply of abalone has increased fivefold since 1970's and farm productions increased explosively from 50 mt to 103,464 mt in the past forty years. Additionally, researchers have recently focused on Abalone given their reported tumor suppression effect. However, despite the valuable features of this marine animal, no genomic information is available for Haliotidae family and related research is still limited.

## Findings

In order to construct the *H.discus hannai* genome, a total of 580G base pairs using Illumina and Pacbio platforms were generated with 322-fold coverage based on the 1.8Gb estimated genome size of *H.discus hannai* using flow cytometry. The final genome assembly consisted of 1.86Gb with 35,450 scaffolds (>2kb). GC content level was 40.51%, and the N50 length of assembled scaffolds was 211kb. We identified 29,449 genes using Evidence Modeler based on the gene information from ab initio prediction, protein homology with known genes and transcriptome evidence of RNA-seq.

## Conclusions

Here we present the first Haliotidae genome, *Haliotis discus hannai*, with sequencing data,

assembly, and gene annotation information. This will be helpful for resolving the lack of genomic information in the Haliotidae family as well as providing more opportunities for understanding gastropod evolution.

**Keywords**

Abalone genome, Halotidae, *Haliotis discus hannai*

# Data description

Abalone is one of the most important marine gastropod molluscs that inhabits various coastal regions of the world. It is well known that abalone habitation impacts algal communications connected with the reef ecosystem, so they are often utilized for ecological research[2]. Among many abalone species, *H.discus hannai* is a widely used ingredient in East Asian cuisine and it is a valuable food resource due to its richness in protein and other nutrients (Figure 1)[3, 4]. It is considered as an important fishery industry animal. The total global supply of abalone has increased fivefold since 1970's. In order to prevent indiscreetly fishing abalones, legal landings from abalone fisheries have made fishery productions decreased gradually from 19,720 mt to 7,486 mt, but have made farm productions increase explosively from 50 mt to 103,464 mt in the past forty years[5]. Additionally, researchers have recently focused on *H.discus hannai* given their reported tumor suppression effect[6-8]. However, despite the valuable features of this marine animal, no genomic information is available. Therefore, the first draft genome in family Haliotidae has the potential to be utilized as a valuable resource for many researchers.

A single wild abalone (*Haliotis discus hannai*) was collected from the brood stock at the Genetic and Breeding Research Center (GBRC) of the National Fisheries Research & Development Institute (NFRDI) on Geoje Island, Korea for sampling. Hemolymph(10ml) was withdrawn from the sole side foot muscle using a syringe. For genomic DNA extraction, hemocytes were harvested from fresh hemolymph by centrifugation at 3000 × rpm for 5 min at 4℃. Genomic DNA was extracted using a DNeasy Animal Mini Kit (Qiagen, Hilden, Germany). A total 39.38 ug of DNA was quantified using the standard procedure of Quant-iT PicoGreen dsDNA Assay Kit (Molecular Probes, Eugene, OR, USA) with Synergy HTX Multi-Mode Reader (Biotek, Winooski, VT, USA). Quality of DNA was also checked using

ND-1000 spectrophotometer (Thermo Scientifc, Wilmington, DE, USA).

For whole genome shotgun sequencing and draft genome assembly, we used multiple sequencing platforms (Illumina Hiseq2000, Nextseq500 and Pacbio RS II) with 7 different libraries. First, two paired-end libraries with insert sizes of 250bp and 350bp were constructed using Illumina TruSeq DNA Sample Prep. Kit (Illumina, San Diego, CA). Mate pair libraries with insert sizes of about 3k, 5k, 8k, and 10k were constructed for scaffolding process using Illumina Nextera mate-pair library construction protocol (Illumina, San Diego, CA). For high-quality genome assembly, long mate pair library with insert size over 40kb is essential. We tried to construct a long mate pair library using 40kb fosmid clone. However, efficiency of fosmid library construction was very low and we could not retain enough amount of clone. Therefore, Pacbio system was employed for final scaffolding process using long read. Pacbio long reads were generated using P6-C4 chemistry of Pacbio RS II system. Detailed information about the constructed library and generated sequencing data is provided in Table 1. Quality control process of generated raw data was conducted for downstream analysis. Quality of raw data was checked using FASTQC[9] and adapter sequences were removed via Trimmomatic[10], for paired-end libraries, and Nxtrim[11], for mate-pair libraries. K-mer frequency analysis of the abalone genome was conducted using a paired-end library with 350bp insert-size and the jellyfish[12] command-line program. The K-mer distribution of the paired-end library provides valuable information about the target genome. As a result, 19-mer distribution of *Haliotis discus hannai* genome was generated (Figure 2). Genome size estimation based on the 19-mer distribution was conducted through "Estimate genome size.pl" code (https://github.com/josephryan/estimate_genome_size.pl/wiki/Estimate-genome-size.pl". The estimated genome size of *H.discus hannai* using 19-mer distribution was about 1.65Gb.

Based on the 19-mer distribution of paired-end reads, there was a second peak located in the half x-axis of the main peak. This result indicates that *H.discus hannai* genome had high heterozygous genetic character or probable DNA contamination from other organisms. Therefore, before genome assembly, raw reads from Hiseq2000, Nextseq500 paired-end and mate pairs were pre-processed by bacterial sequences, duplicates, and ambiguous nucleotides. To remove the contaminant sequence, clean reads without adapter and low quality bases were mapped to bacterial and ocean metagenome databases downloaded from NCBI by applying the default setting run (-s 0.8 –l 0.5) of clc_mapper (http://www.clcbio.com). After that, duplicates and ambiguous nucleotides were filtered out using clc_remove_duplicates (http://www.clcbio.com). The resulting high-quality sequences were used in subsequent assembly. Error correction and initial contig assembly was conducted using clc_assembler within the CLC Assembly Cell (http://www.clcbio.com/products/clc-assembly-cell/) software pipeline. Scaffolds were then built using the mate-pairs and Pacbio RS II reads sequentially by SSPACE[13] and PBJelly2[14]. After scaffolding, we iteratively conducted gap filling process using Gapcloser[15] using -l 155 and -p 31 parameter option. Summary statistics for final assembly is provided in Table 2.

Before conducting gene prediction using the assembled sequence, repeat elements were identified using RepeatMasker[16] with Repbase[17]. RepeatModeler, which includes RECON[18], RepeatScout[19] and TRF[20], was used to create a custom database of *H.discus hannai*. After custom library construction, RepeatMasker with RMBlast was used for each genome with 'no_is' option, using repeat libraries from RepeatModeler and Repbase. Identified mobile elements are summarized in Table 3. Identified repeat elements were parsed for identifying more detail information using a perl code named "One code to find them all"[21] and Figure S1 shows the proportion of each mobile element. The genome size of *H.*

*discus hannai* was 1.86 Gb, and this is the biggest genome among known gastropods. It is 5.31 and 2.02 times larger than genomes size of *L.gigantea* (0.35 Gb) and *A.californica* (0.92 Gb) in the same Gastropoda class. In animals, the increase of genome size is commonly driven by transposable element, and this is a known genetic adaption mechanism to stressful environments[22]. Therefore, we conducted comparative analysis of repeat element against *L.gignatea*, a same marine gastropod with large genome size difference with that of *H.discus hannai*, to identify the reason for this large difference. Figure 3a shows the amount and proportion of identified repeat element from two marine gastropods. The proportion of identified total repeat elements in *H.discus hannai* and *L.gigantea* is respectively 30.76% and 22.25% of genome size, and a total amount of identified repeat elements in *H.discus hannai* genome is almost six times larger than that of *L.gigantea* same as genome size. Such linear relationship between genome size and the total proportion of repeat elements is consistent with a previous study[23]. The proportion, copy number and divergence of each mobile element were identified and compared (Figure S2-6) for a deeper understanding of mobile elements in two species. From the comparison, a notable finding has been observed on mobile elements: DNA transposable element, a Class II transposable element, exists in diverse forms in both species; however, retrotransposon element, a Class I transposable element, is much more abundant in *H.discus hannai* genome than in *L.gigantea* genome. Especially, the number of a non-LTR retrotransposon called LINE Element was exceptionally high. Figure 3-b illustrates the difference between the two species, using two signature mobile elements (*H.discus hannai*: LINE/I, DNA/TcMar-Tc1, *L.gigantea*: DNA/RC, DNA/Maverick) in each genome. DNA/RC and DNA/Maverick, two major mobile elements in *L.gigantea* genome, are observed in *H.discus* in somewhat similar distribution. On the other hand, the two signature mobile elements of *H.discus hannai* genome, LINE/I and DNA/TcMar-Tc1, are specifically abundant in *H.discus hannai* and seems to have expanded

recently diverged compared to other elements. In sum, species specificity can be inferred from the distinctive patterns of repeat element expansion between the two species and the increased genome size of *H.discus hannai* may be associated with the non-LTR elements (especially LINE/I) contribution, in parallel to the human genome[23].

Genes were predicted through three different algorithms: *ab initio*, RNA-seq transcript based, and protein homology-based. For RNA-seq transcript based prediction, transcriptome data from six organ tissues (Table 4) were aligned to the assembled genome sequence using Tophat[24], and transcript structure was predicted through Cufflinks[25]. The homology-based method employs complete protein sequences from diverse taxonomical genomes, which is fit to our model. For *Haliotis discus hannai,* the following 8 species were utilized: *Lottia gigantea, Crassostrea gigas, Aplysia california, Strongylocentrtus purpuratus, Branchiostoma floridae, Danio rerio, Oncorhynchus mykiss and Homo sapiens*. Those protein sequences were aligned to the *Haliotis discus hannai* genome using TBASTN (E-value ≤ 1E-4)[26]. Next, the homologous genome sequences were aligned to the matched proteins using Exonerate[27] to predict the accurate spliced alignments. Table 5 summarizes the alignment results of known proteins in various species. For *ab initio* gene prediction, Augustus[28] was trained using RNA-seq data and known proteins by using the complete transcriptome as training matrix for HMM. Fgenesh[29] and Geneid[30] were also used. The parameters used and the number of predicted genes is provided in Table 6. Gene prediction data from each method was combined using EVM (Evidence Modeler)[31] to build a consensus gene set for the abalone genome. All gene models were converted to EVM compatible GFF3 format and merged to a consensus gene set. After consensus gene annotation was generated from EVM, manual curation was conducted for abandon genes from EVM to build a final consensus gene set of *H.discus hannai*. Manual curation was

performed based on the genomic DNA mapping position of the RNA-seq sequence and the protein sequence of the related species. In order to determine the exon-intron edge of the gene, the genome mapping information of the transcriptome sequence was firstly reflected, and if not, the mapping information of the protein sequence of the related species was referred to secondarily to confirm the gene model. Finally, genes that were not translated into protein sequences in the final gene model were removed. A total of 29,449 genes were predicted in the *H.discus hannai* genome and summary statistics for the consensus gene set is provided in Table 7. To evaluate the quality of *H.discus hannai* draft genome, we conducted paired-end read remapping and BUSCO (Benchmarking Universal Single-Copy Orthologs) analysis. 94.89% of paired-end reads with 350bp insert size were successfully mapped to the assembled genome and assembled genome contains 609 complete and 130 fragmented genes in BUSCO analysis. The detailed information of BUSCO analysis is summarized in Table 8.

In summary, here we report the first annotated Haliotidae genome of *H.discus hannai* based on various genetic evidence. We expect that the_*H.discus hannai genome* presented here, which is the first genome to be sequenced in the family Haliotidae, will provide useful genomic information for many researchers. *Haliotis discus hannai* is a cold-water abalone breed that have difficulties dealing with the change in their inhabitable latitude, which is due to global warming and the resulting increase in the rate of sudden perishing. Genomic information of abalone is essential information which can be used for genetic breeding to improve productivity and genetic engineering for the heat resistance breed. It can also provide valuable information for future genomic studies because only limited genome information about marine animals and mollusks is currently available. Evolutionary signatures recorded in abalone genome can be identified through future comparative genomic studies and we expect our result will provide more insight into Haliotidae and marine mollusk

evolution.

## Availability of supporting data

Raw data is available in project accession PRJNA317403 in the NCBI database. Further supporting data can be found in the *GigaScience* GigaDB [32].

## List of abbreviations

GBRC - Genetic and Breeding Research Center

NFRDI - National Fisheries Research & Development Institute

EVM - Evidence Modeler

BUSCO - Benchmarking Universal Single-Copy Orthologs

## Competing interests

All authors report no competing interests.

## Authors contributions

Sampling - Bo-Hye Nam, Young-Ok Kim, Dong-Gyun Kim

Sequencing - Bo-Hye Nam, Hee Jeong Kong, Woo-Jin Kim, Jeong-Ha Kang, Ji-Young Moon, Choul Ji Park, Duk Kyung Kim

Genome assembly - Bo-Hye Nam, Woori Kwak, Jae Woong Yu, Joon Yoon, SaetByeol Lee, Samsun Sung, Chul Lee, Sojeong Ka, Kelsey Caetano-Anolles

Repeat element analysis - Woori Kwak, Minseok Seo, Kwondo Kim

Gene prediction - Woori Kwak, Younhee Shin, Myunghee Jung, Byeong-Chul Kang, Ga-hee Shin

Funding and experimental design – Jung Youn Park, Cheul Min An, Seoae Cho, Heebal Kim

## Acknowledgements

This work was supported by a grant from the National Institute of Fisheries Science (R2016024).

# References


1. Appeltans, W., et al., *World register of marine species.* Accessed online: http://www.marinespecies.org (accessed on 28 February 2014), 2012.
2. Hamer, P., et al., *Understanding the ecological role of abalone in the reef ecosystem of Victoria.* Fisheries Research Report, 2010.
3. Elliott, N.G., *Genetic improvement programmes in abalone: what is the future?* Aquaculture research, 2000. **31**(1): p. 51-59.
4. Gordon, H.R. and P.A. Cook, *World abalone fisheries and aquaculture update: supply and market dynamics.* Journal of shellfish research, 2004. **23**(4): p. 935-940.
5. Cook, P.A., *The worldwide abalone industry.* Modern Economy, 2014. **5**(13): p. 1181.
6. Suleria, H.R., et al., *Therapeutic potential of abalone and status of bioactive molecules: A comprehensive review.* Critical reviews in food science and nutrition, 2015.
7. Lim, S.Y., *Cytotoxic and Antioxidant Activities of Abalone (Haliotis discus hannai) Extracts.* Journal of life science, 2014. **24**(7): p. 737-742.
8. Lee, C.-G., et al., *Abalone visceral extract inhibit tumor growth and metastasis by modulating Cox-2 levels and CD8+ T cell activity.* BMC complementary and alternative medicine, 2010. **10**(1): p. 1.
9. Andrews, S., *FastQC a quality-control tool for high-throughput sequence data* http://www.bioinformatics.babraham.ac.uk/projects/fastqc/, 2014.
10. Bolger, A.M., M. Lohse, and B. Usadel, *Trimmomatic: a flexible trimmer for Illumina sequence data.* Bioinformatics, 2014: p. btu170.
11. O'Connell, J., et al., *NxTrim: optimized trimming of Illumina mate pair reads.* Bioinformatics, 2015. **31**(12): p. 2035-2037.
12. Marçais, G. and C. Kingsford, *A fast, lock-free approach for efficient parallel counting of occurrences of k-mers.* Bioinformatics, 2011. **27**(6): p. 764-770.
13. Boetzer, M., et al., *Scaffolding pre-assembled contigs using SSPACE.* Bioinformatics, 2011. **27**(4): p. 578-579.
14. English, A.C., et al., *Mind the gap: upgrading genomes with Pacific Biosciences RS long-read sequencing technology.* PloS one, 2012. **7**(11): p. e47768.
15. Luo, R., et al., *SOAPdenovo2: an empirically improved memory-efficient short-read de novo assembler.* GigaScience, 2012. **1**(1): p. 1-6.
16. Tarailo-Graovac, M. and N. Chen, *Using RepeatMasker to identify repetitive elements in genomic sequences.* Current Protocols in Bioinformatics, 2009: p. 4.10. 1-4.10. 14.
17. Jurka, J., et al., *Repbase Update, a database of eukaryotic repetitive elements.* Cytogenetic and genome research, 2005. **110**(1-4): p. 462-467.
18. Bao, Z. and S.R. Eddy, *Automated de novo identification of repeat sequence families in sequenced genomes.* Genome Research, 2002. **12**(8): p. 1269-1276.

19. Price, A.L., N.C. Jones, and P.A. Pevzner, *De novo identification of repeat families in large genomes.* Bioinformatics, 2005. **21**(suppl 1): p. i351-i358.

20. Benson, G., *Tandem repeats finder: a program to analyze DNA sequences.* Nucleic acids research, 1999. **27**(2): p. 573.

21. Bailly-Bechet, M., A. Haudry, and E. Lerat, *"One code to find them all": a perl tool to conveniently parse RepeatMasker output files.* Mobile DNA, 2014. **5**(1): p. 1.

22. Chénais, B., et al., *The impact of transposable elements on eukaryotic genomes: from genome size increase to genetic adaptation to stressful environments.* Gene, 2012. **509**(1): p. 7-15.

23. Kidwell, M.G., *Transposable elements and the evolution of genome size in eukaryotes.* Genetica, 2002. **115**(1): p. 49-63.

24. Trapnell, C., L. Pachter, and S.L. Salzberg, *TopHat: discovering splice junctions with RNA-Seq.* Bioinformatics, 2009. **25**(9): p. 1105-1111.

25. Trapnell, C., et al., *Differential gene and transcript expression analysis of RNA-seq experiments with TopHat and Cufflinks.* Nature protocols, 2012. **7**(3): p. 562-578.

26. Altschul, S.F., et al., *Gapped BLAST and PSI-BLAST: a new generation of protein database search programs.* Nucleic acids research, 1997. **25**(17): p. 3389-3402.

27. Slater, G.S. and E. Birney, *Automated generation of heuristics for biological sequence comparison.* BMC bioinformatics, 2005. **6**(1): p. 31.

28. Stanke, M., et al., *Using native and syntenically mapped cDNA alignments to improve de novo gene finding.* Bioinformatics, 2008. **24**(5): p. 637-644.

29. Solovyev, V., et al., *Automatic annotation of eukaryotic genes, pseudogenes and promoters.* Genome Biol, 2006. **7**(Suppl 1): p. S10.

30. Blanco, E., G. Parra, and R. Guigó, *Using geneid to identify genes.* Current protocols in bioinformatics, 2007: p. 4.3. 1-4.3. 28.

31. Haas, B.J., et al., *Automated eukaryotic gene structure annotation using EVidenceModeler and the Program to Assemble Spliced Alignments.* Genome biology, 2008. **9**(1): p. R7.

32. Nam B, Kwak W, Kim Y, Kim D, Kong HJ, Kim W, Kang J, Park JY, An CM, Moon J, Park CJ, Yu JW, Yoon J, Seo M, Kim K, Kim DK, Lee S, Sung S, Lee C, Shin Y, Jung M, Kang B, Shin G, Ka S, Caetano-Anolles K, Cho S, Kim H: Supporting data for "Genome sequence of pacific abalone (Haliotis discus hannai): the first draft genome in family Haliotidae" GigaScience Database. 2017. http://dx.doi.org/10.5524/100281

# Tables

Table 1. Summary statistics of generated whole genome shotgun sequencing data.

| Library Name | Library Type | Insert Size | Platform | Read Length | No. Read | Total bp |
|---|---|---|---|---|---|---|
| 250bp | Paired-end | 250 | Nextseq500 | 150 | 876,529,480 | 131,440,418,087 |
| 350bp | Paired-end | 350 | Hiseq2000 | 101 | 1,413,620,786 | 142,775,699,386 |
| 3k | Mate-pair | 3,000 | Nextseq500 | 150 | 580,064,464 | 85,689,154,056 |
| 5k | Mate-pair | 5,000 | Nextseq500 | 150 | 468,432,888 | 69,966,139,205 |
| 8k | Mate-pair | 8,000 | Nextseq500 | 150 | 335,132,792 | 50,109,845,012 |
| 10k | Mate-pair | 10,000 | Nextseq500 | 150 | 569,376,096 | 85,080,237,236 |
| 20k | P6-C4 | 20,000 | Pacbio RS II | 10,094 (average) | 1,573,020 | 15,879,626,978 |
| **Total** | | | | | | 580,941,119,960 |

Table 2. Summary statistics for the *Haliotis discus hannai* draft genome (>2kb).

| **Assembled Genome** | |
|---|---|
| Size(1n) | 1.80 Gb |
| GC level | 40.51% |
| No. scaffolds | 35,450 |
| N50 of scaffolds (bp) | 211,346 |
| N bases in scaffolds (%) | 116 Mb (6.45%) |
| Longest(shortest) scaffolds (bp) | 2,207,537 (2,000) |
| Average scaffold Length (bp) | 50,870.65 |

| **Repeat Element** | No. Element | Length (%) |
|---|---|---|
| SINE | 284,485 | 96,155,199 (5.11%) |
| LINE | 700,245 | 160,387,248 (8.53%) |
| LTR element | 383,770 | 55,149,794 (2.93%) |
| DNA element | 58,022 | 14,563,432 (0.77%) |
| Small RNA | 20,997 | 1,537,853 (0.08%) |
| Simple repeat | 161,246 | 32,547,245 (1.73%) |
| Low complexity | 326,399 | 21,446,303 (1.14%) |
| Unclassifed | 1,522,272 | 265,603,066 (14.1%) |

Table 3. Summary of identified repeat elements in the *Haliotis discus hannai* genome.

Table 4. Summary statistics of generated transcriptome data for six organ tissues using Illumina platform.

| Library Name | Library Type | Platform | Read Length | No. Read | Total bp |
|---|---|---|---|---|---|
| Blood | Paired-end | Hiseq2000 | 101 | 53,525,950 | 5,406,120,950 |
| Digestive duct | Paired-end | Hiseq2000 | 101 | 56,485,666 | 5,705,052,266 |
| Gill | Paired-end | Hiseq2000 | 101 | 66,415,882 | 6,708,004,082 |
| Hepatopancreas | Paired-end | Hiseq2000 | 101 | 58,467,176 | 5,905,184,776 |
| Mantle | Paired-end | Hiseq2000 | 101 | 65,741,776 | 6,639,919,376 |
| Ovary | Paired-end | Hiseq2000 | 101 | 60,997,100 | 6,160,707,100 |
| Total | | | | | 36,524,988,550 |

| Species | Type | Element | Total count | Count/ Gene | Total length, bp | Mean length, Bp | Genome Coverage % |
|---|---|---|---|---|---|---|---|

Table 5. Summary statistics of protein alignment using tBlastn for protein based evidence gene structure.

| | | | | | | | |
|---|---|---|---|---|---|---|---|
| *Homo sapiens* | Protein (69,002) | Transcript | 18,792 | | 109,068,639 | 5,803.99 | 5.80 |
| | | Exon | 77,320 | 4.11 | 12,667,395 | 163.83 | 0.67 |
| *Danio rerio* | Protein (42,474) | Transcript | 11,605 | | 68,796,463 | 5,928.17 | 3.66 |
| | | Exon | 47,300 | 4.08 | 7,978,167 | 168.67 | 0.42 |
| *Oncorhynchus mykiss* | Protein (53,876) | Transcript | 15,901 | | 55,043,032 | 3,461.61 | 2.93 |
| | | Exon | 46,040 | 2.90 | 7,567,059 | 164.36 | 0.40 |
| *Lottia gigantea* | Protein (23,851) | Transcript | 29,345 | | 177,851,531 | 6,060.71 | 9.47 |
| | | Exon | 118,165 | 4.03 | 20,583,999 | 174.20 | 1.10 |
| *Crassostrea gigas* | Protein (28,027) | Transcript | 32,978 | | 231,175,282 | 7,009.98 | 12.30 |
| | | Exon | 140,784 | 4.27 | 23,649,828 | 167.99 | 1.26 |
| *Aplysia californica* | Protein (29,096) | Transcript | 10,570 | | 67,396,621 | 6,376.22 | 3.59 |
| | | Exon | 45,737 | 4.33 | 7,797,503 | 170.49 | 0.42 |
| *Strongylocentrotus purpuratus* | Protein (38,730) | Transcript | 9,116 | | 46,270,640 | 5,075.76 | 2.46 |
| | | Exon | 34,572 | 3.79 | 5,627,082 | 162.76 | 0.30 |
| *Branchiostoma floridae* | Protein (58,493) | Transcript | 27,438 | | 125,307,206 | 4,566.92 | 6.67 |
| | | Exon | 92,426 | 3.37 | 15,483,164 | 167.52 | 0.82 |

Table 6. Summary statistics for ab initio gene prediction results using various programs and parameters.

| Program | Matrix | Element | Total count | Count/Gene | Total length, bp | Mean length, bp | Genome Coverage % |
|---|---|---|---|---|---|---|---|
| | Custom parameter (RNAseq) | Gene | 88,825 | 3.92 | 367,066,732 | 4,132.47 | 19.54 |
| | | CDS | 348,528 | | 76,388,076 | 219.17 | 4.07 |
| Augustus | Custom parameter (*H.discus hannai* IsoSeq) | Gene | 90,396 | 4.11 | 395,511,710 | 4,375.32 | 21.05 |
| | | CDS | 371,487 | | 78,508,401 | 211.34 | 4.18 |
| | Custom parameter (H.discus discus IsoSeq) | Gene | 84,322 | 3.97 | 346,455,180 | 4,108.72 | 18.44 |
| | | CDS | 335,103 | | 72,527,841 | 216.43 | 3.86 |
| | Custom parameter (BUSCO) | Gene | 111,058 | 4.24 | 626,749,935 | 5,643.45 | 33.36 |
| | | CDS | 470,839 | | 84,333,972 | 179.11 | 4.49 |
| | Custom parameter (CEGAM) | Gene | 76,504 | 4.95 | 393,121,657 | 5,138.58 | 20.92 |
| | | CDS | 378,485 | | 63,424,677 | 167.58 | 3.38 |
| | Custom parameter (Protein) | Gene | 22,420 | 3.43 | 184,289,721 | 8,219.88 | 9.81 |
| | | CDS | 76,848 | | 20,291,739 | 264.05 | 1.08 |
| Fgenesh | Custom parameter | Gene | 184,051 | 3.46 | 1,366,924,540 | 7,426.88 | 72.75 |
| | | CDS | 636,568 | | 98,055,591 | 154.04 | 5.22 |
| Geneid | *Ciona intestinalis* | Gene | 789,540 | 1.41 | 436,990,370 | 553.47 | 23.26 |
| | | CDS | 1,112,959 | | 140,976,492 | 126.67 | 7.50 |

Table 7. Summary statistics for the consensus gene set of *Haliotis discus hannai* genome.

| Element | No. elements | Exon/transcript | Avg. length | Total length | Genome coverage |
|---|---|---|---|---|---|

| | | | | | |
|---|---|---|---|---|---|
| Gene | 29,449 | - | 2,705 | 79,661,536 | 4.2 % |
| Exon | 74,745 | 2.54 | 280 | 20,985,298 | 1.1 % |
| Intron | 45,296 | 1.54 | 1,295 | 58,676,238 | 3.1 % |

Table 8. Summary statistics of Benchmarking Universal Single-Copy Orthologs (BUSCO) analysis for *Haliotis discus hannai* genome based on Metazoans DB.

| **Categories** | **#Genes** | **Percentage** |
|---|---|---|
| Complete Single-Copy BUSCOs | 609 | 72.2% |
| Complete Duplicate BUSCOs | 48 | 5.7% |
| Fragmented BUSCOs | 130 | 15.4% |

| | | |
|---|---|---|
| Missing BUSCOs | 104 | 12.3% |

# Figures

**Figure 1. Example of a *Haliotis discus hannai*, the pacific abalone.**

**Figure 2. 19-mer distribution of using jellyfish with 350bp paired-end whole genome sequencing data.**

**Figure 3. Repeat element information of *H.discus hannai* compared to *L. gigantean*. a**, Total amount and ratio of identified repeat element classified into 8 classes (DNA, LINE, SINE, LTR, Low complexity, Satellite, Simple repeat and Unknown) from each genome. **b**, Distribution of gene copy number of the two highly possessed repeat elements in each genome based on the divergence. Heat maps indicate the total amount of repeat element divided into 20 levels based on the divergence.

Figure 1. Abalone Picture

Click here to download Figure Abalone.jpg

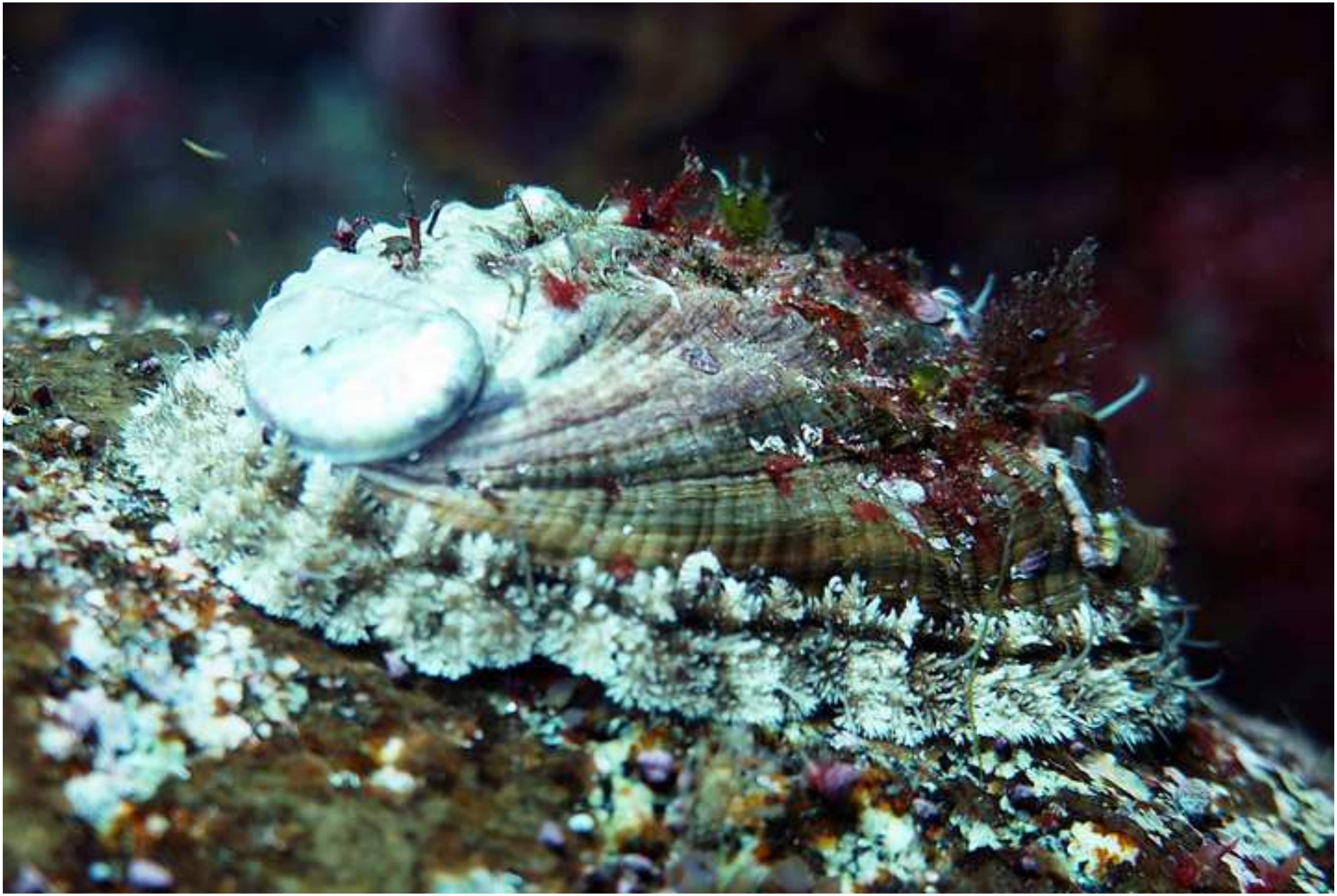

Click here to download Figure Figure1.pdf

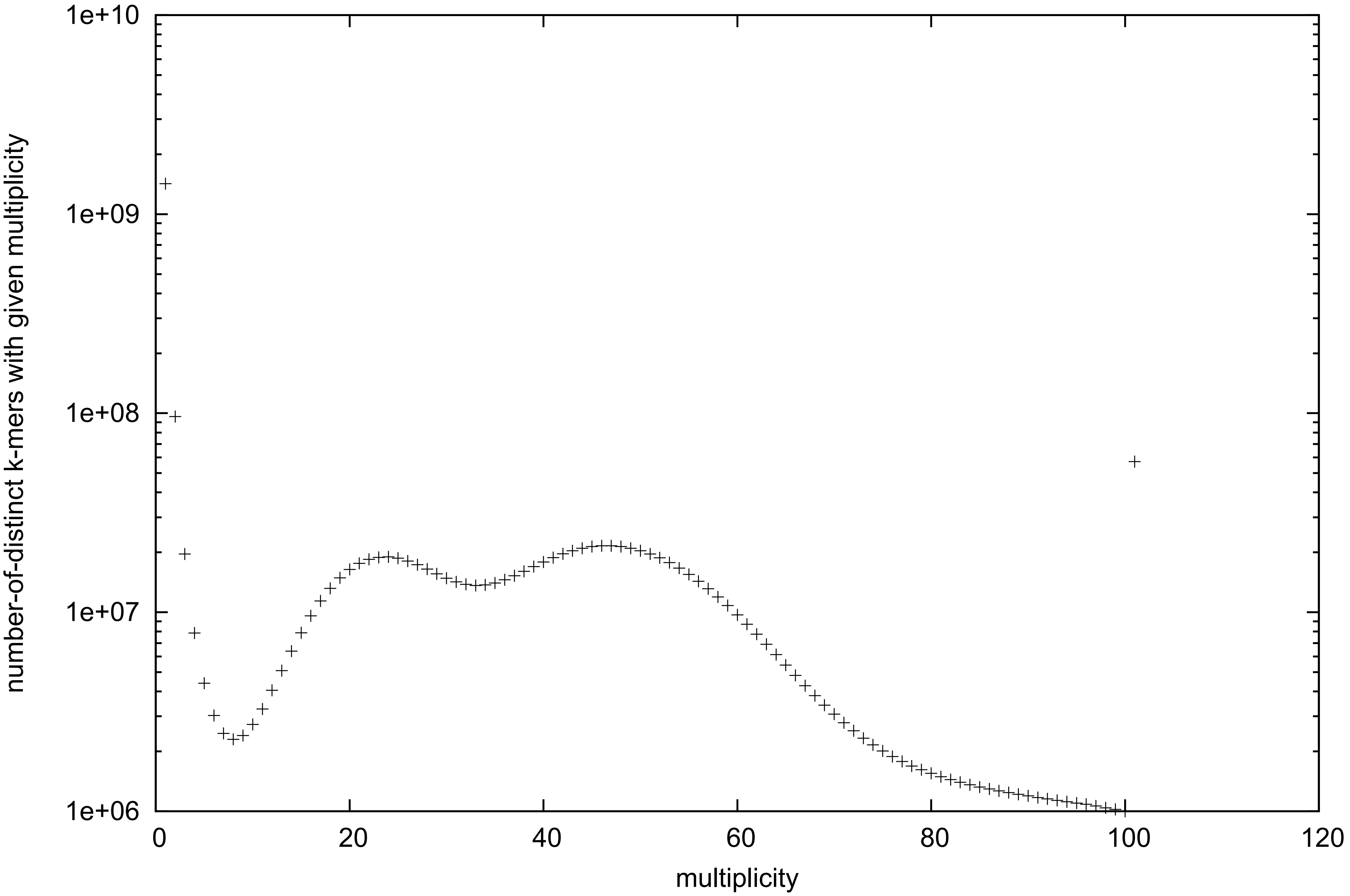

Click here to download Figure Figure2.pdf

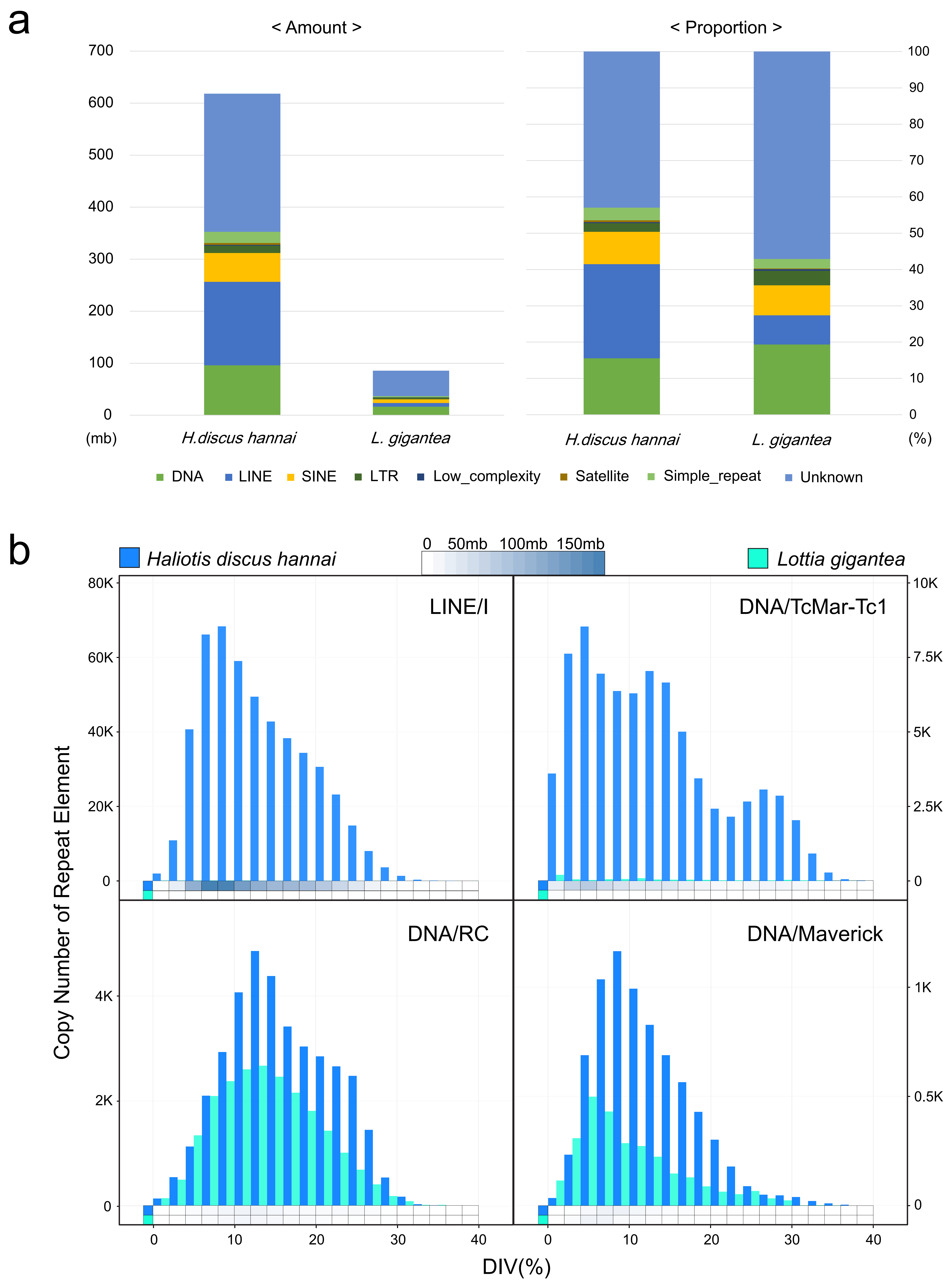

Click here to access/download
**Supplementary Material**
Supplementary_Information.docx

Click here to access/download
**Supplementary Material**
Response_to_Reviewers.doc

Personal Cover Click here to download Personal Cover Cover_letter.docx

# SEOUL NATIONAL UNIVERSITY

**Laboratory of Bioinformatics and Population Genetics**

**Heebal Kim, Ph.D.**
**Professor**

February, 2, 2017

Editor in Chief, *GIGA SCIENCE*

Dear. Editor,

We are pleased to submit our revised manuscript entitled "Genome sequence of pacific abalone (Haliotis discus hannai): the first draft genome in family Haliotidae." by Nam et al, for publication as a data note in *GIGA SCIENCE.*

Followed the directions of editor and reviewer #3, we revised our manuscript and addressed all the issues. We hope our revised manuscript meet the high-quality standard of *GIGA SCIENCE*. Feel free to get in touch with me if you have any questions.

Yours sincerely,

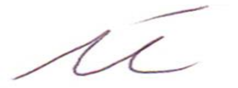

Heebal Kim on behalf of all the authors

Laboratory of Bioinformatics and Population Genetics, Department of Agricultural Biotechnology, Seoul National University, San 56-1, Sillim-dong, Gwanak-gu, Seoul 151-742, Korea Phone: 82-2-880-4803, Fax: 82-2-883-8812, Email: heebal@snu.ac.kr

**Dept of Agricultural Biotechnology**
**Seoul National University**
**San 56-1 Daehak-dong, Gwanak-gu, Seoul 151-742**
**KOREA**

**T +82 2 880 4803**
**F +82 2 883 8812**
**heebal@snu.ac.kr**
