# Supplementary material for: Genome sequence of pacific abalone (Haliotis discus hannai): the first draft genome in family Haliotidae
Source: Gigascience. 2017 Mar 7;6(5):1–8. doi: 10.1093/gigascience/gix014 (PMC5439488; doi:10.1093/gigascience/gix014)

## Figure S1. Tree map for sum of repeat element for *Haliotis discus hannai*.


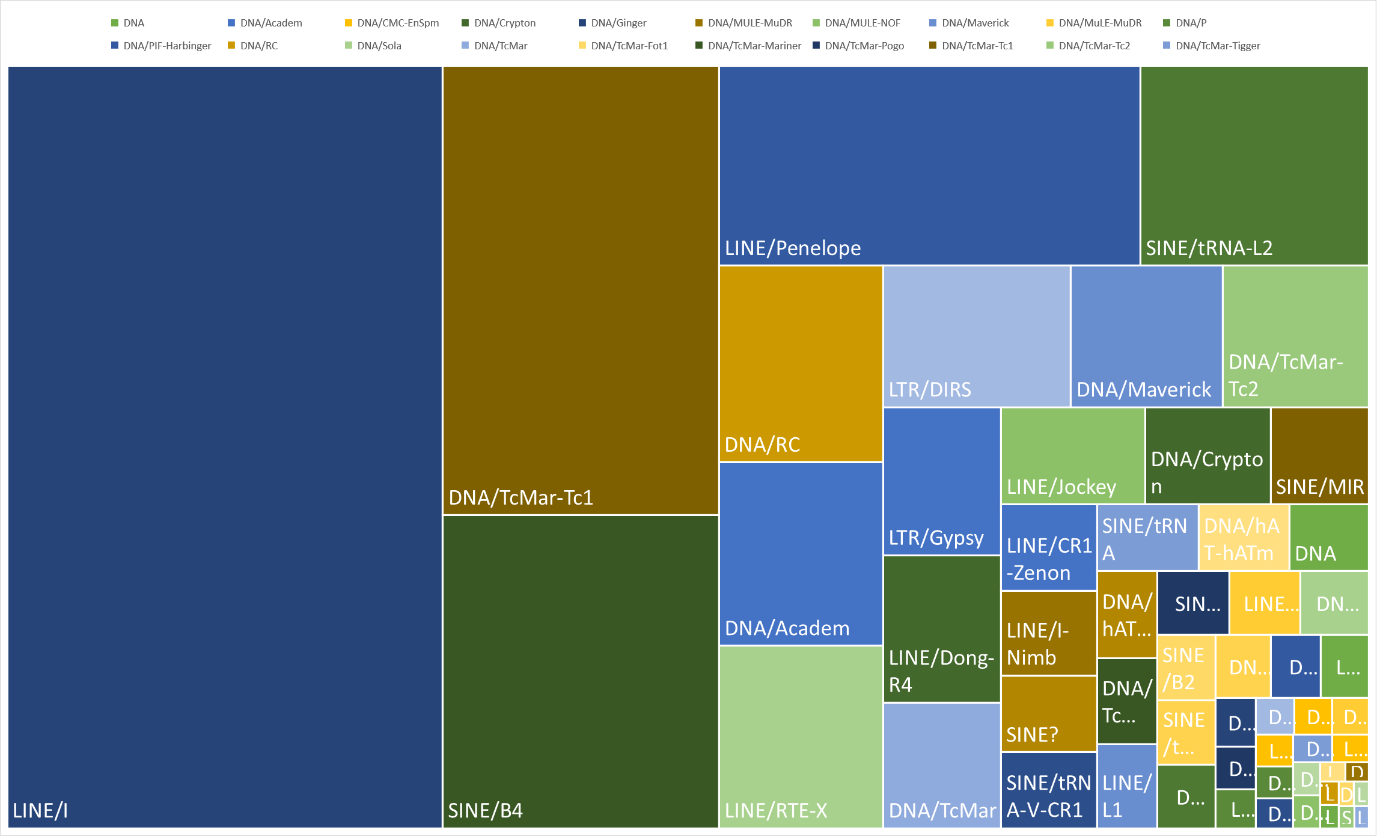


## Figure S2. Comparison of SINE element distribution of *Haliotis discus hannai* and *Lottia gigantea.*


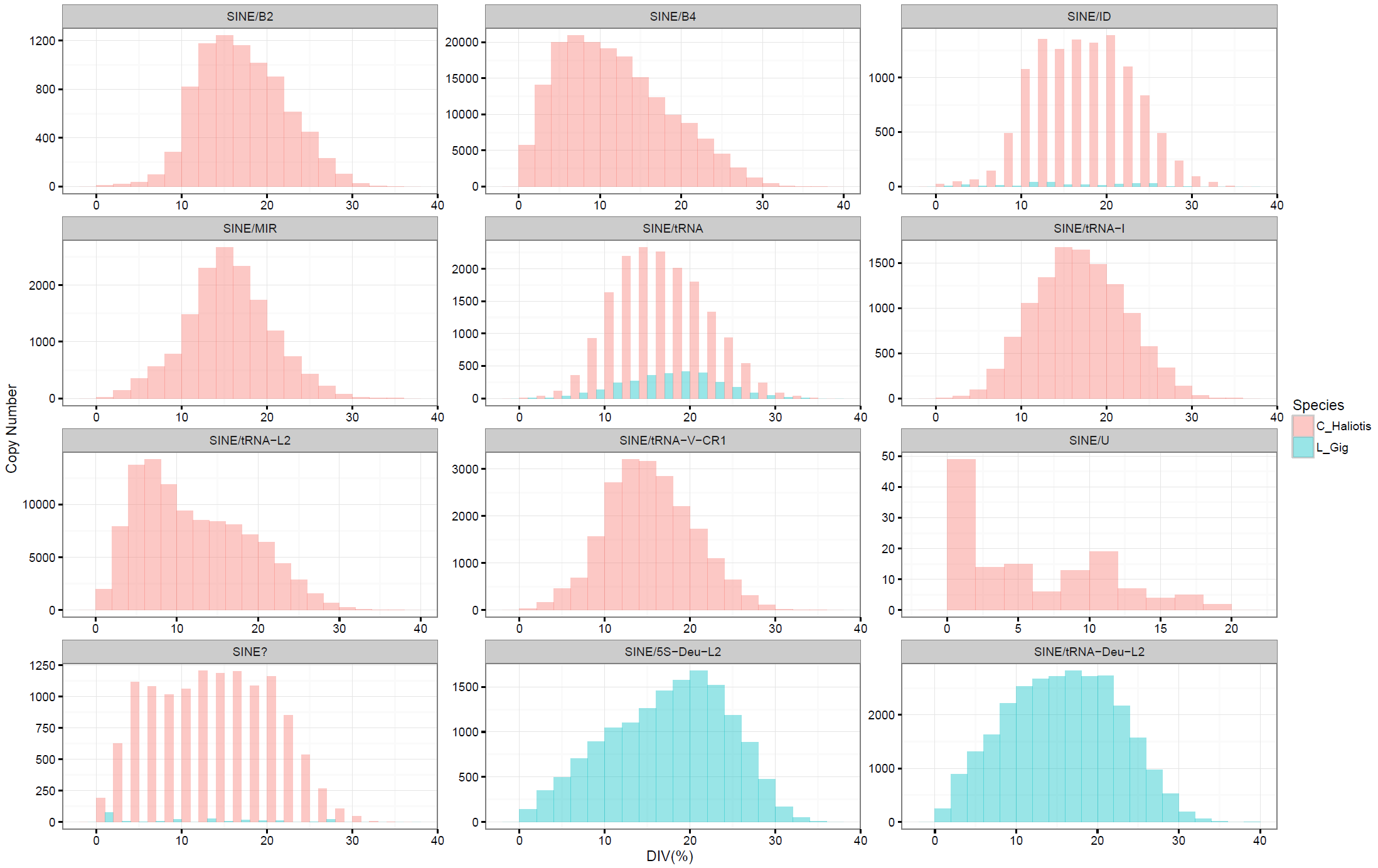


## Figure S3. Comparison of LINE element distribution of *Haliotis discus hannai* and *Lottia gigantea.*


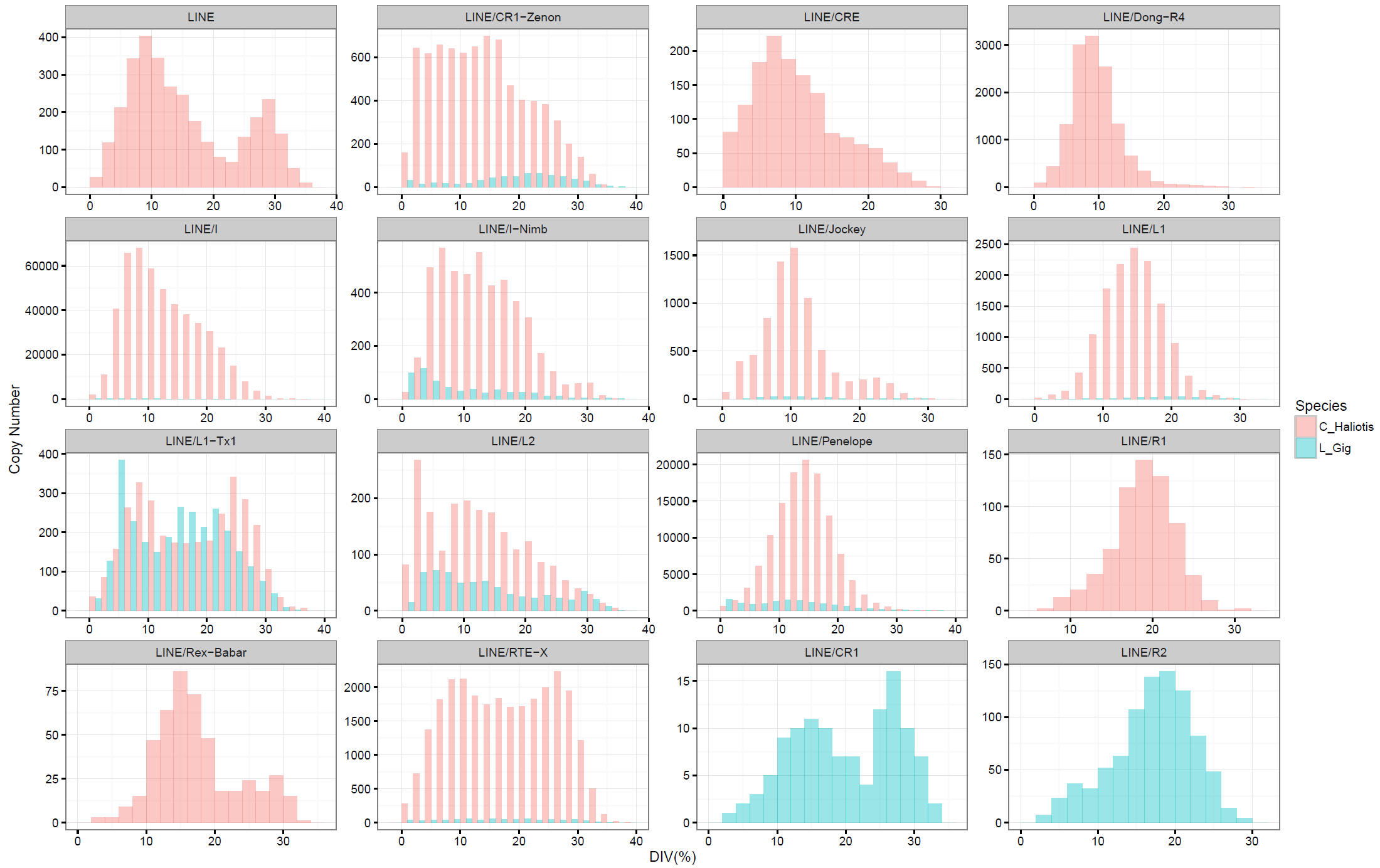


##
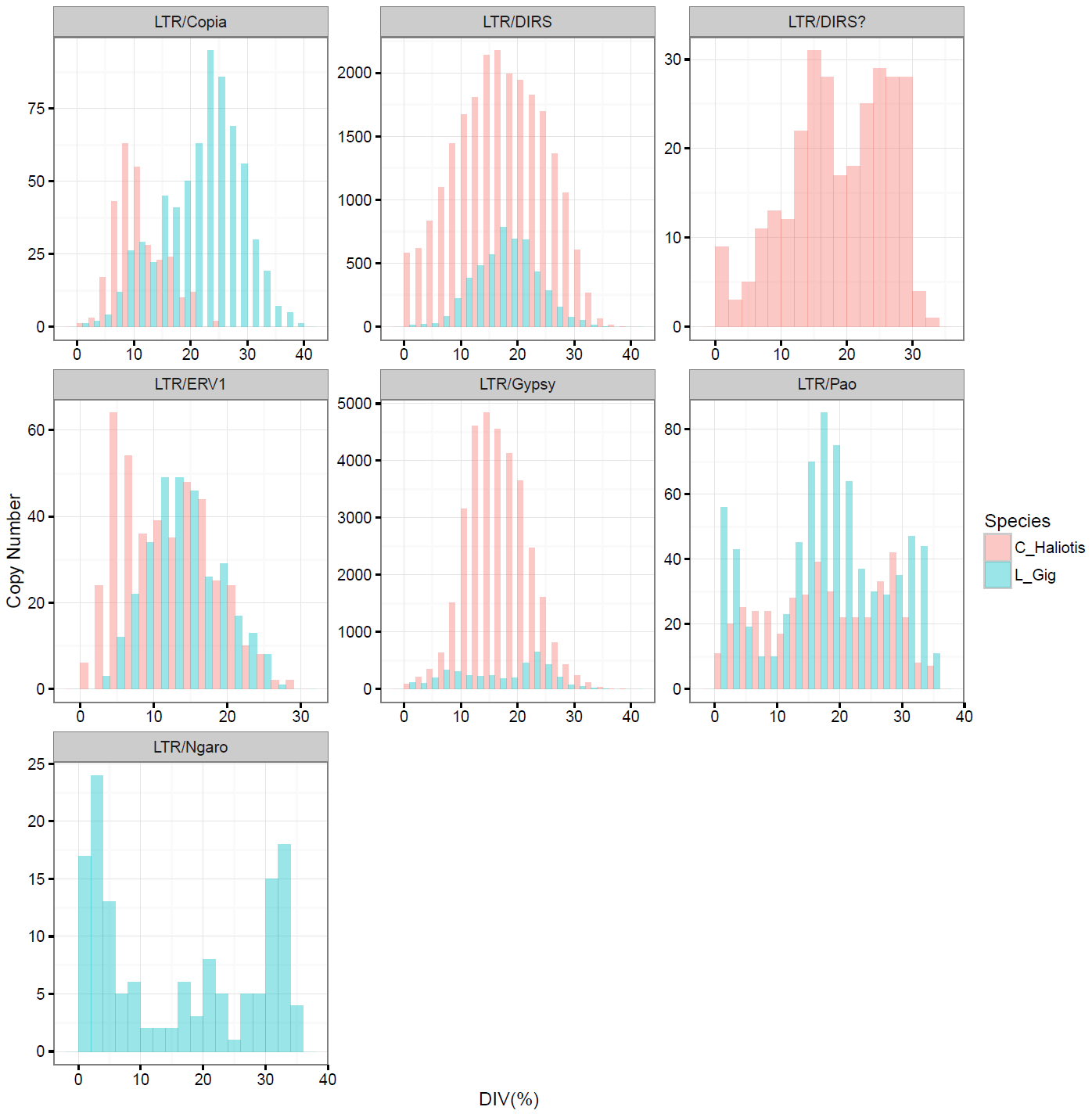
Figure S4. Comparison of LTR element distribution of *Haliotis discus hannai* and *Lottia gigantea.*

## Figure S5. Comparison of DNA transposon element distribution of *Haliotis discus hannai* and *Lottia gigantea.*


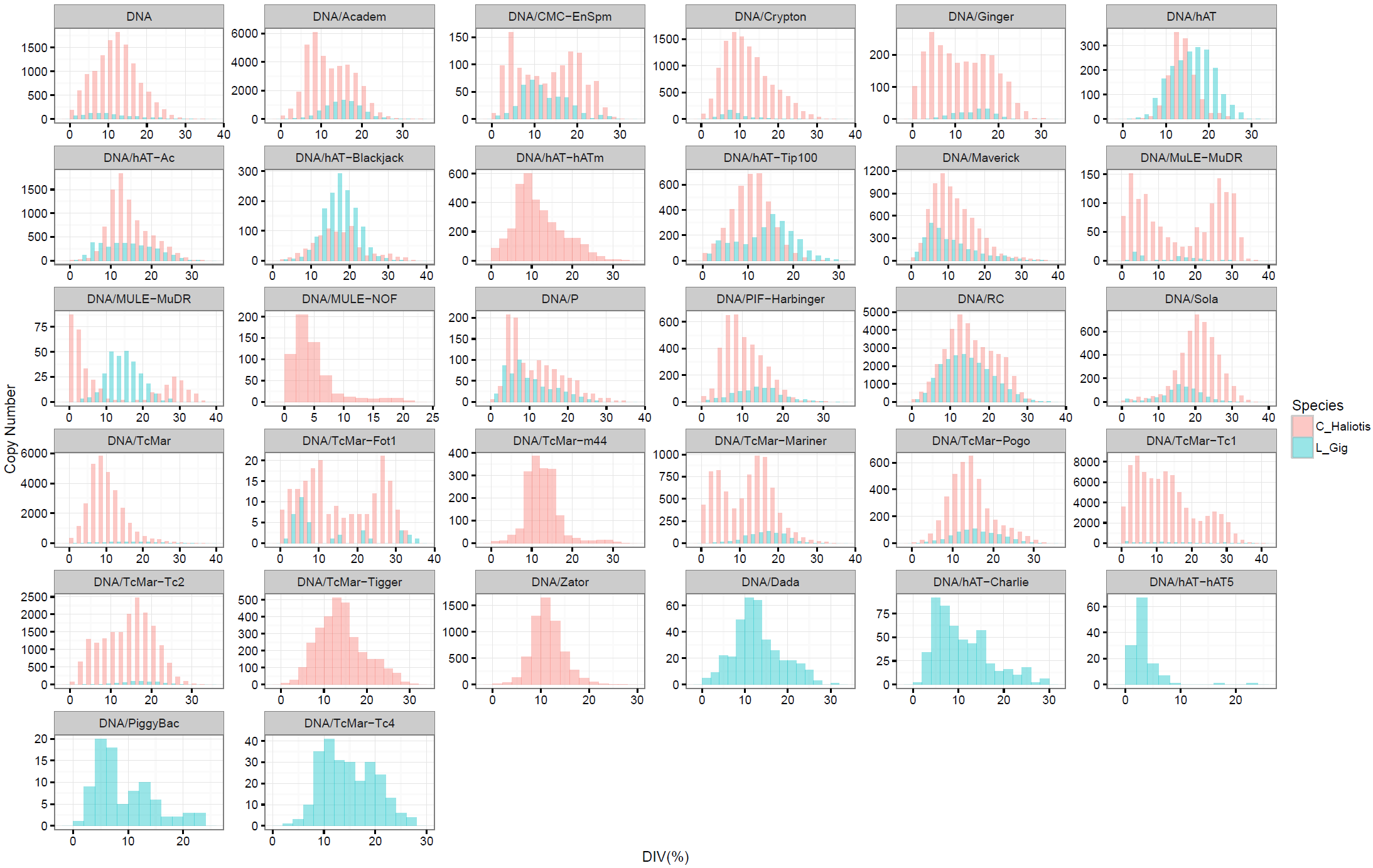

Supplement: Supplemental material — Figure S1. Tree map for sum of repeat element for H. discus hannai. Figure S2. Comparison of SINE element distribution of H. discus hannai and L. gigantea. Figure S3. Comparison of LINE element distribution of H. discus hannai and L. gigantea. Figure S4. Comparison of LTR element distribution of H. discus hannai and L. gigantea. Figure S5. Comparison of DNA transposon element distribution of H. discus hannai and L. gigantea. [file gix014_Supp.docx]
