# Supplementary material for: Genome sequence of pacific abalone (Haliotis discus hannai): the first draft genome in family Haliotidae
Source: Gigascience. 2017 Mar 7;6(5):1–8. doi: 10.1093/gigascience/gix014 (PMC5439488; doi:10.1093/gigascience/gix014)
Supplement: Response_to_reviewer_comments_Original_Manuscript.pdf [file gix014_Response_to_reviewer_comments_Original_Manuscript.pdf]

We attached "Respond to Reviewer document" seperately for respsonse.
