# Supplementary material for: Genome sequence of pacific abalone (Haliotis discus hannai): the first draft genome in family Haliotidae
Source: Gigascience. 2017 Mar 7;6(5):1–8. doi: 10.1093/gigascience/gix014 (PMC5439488; doi:10.1093/gigascience/gix014)
Supplement: Reviewer_3_Report_(Original_Submission).pdf [file gix014_Reviewer_3_Report_(Original_Submission).pdf]

Reviewer Report

Title: "Genome sequence of pacific abalone (Haliotis discus hannai): the first draft genome in family Haliotidae."

Version: Original Submission **Date:** 1/15/2017

Reviewer name: Mathew Jenny

Reviewer Comments to Author:

The manuscript entitled "Genome sequence of Pacific abalone (Haliotis discus hannai): the first draft genome in family Haliotidae" by Nam et al, provides the initial results of the genome assembly for what is an ecologically and economically important molluscan species. The completion of this genome assembly and annotation will provide additional new resources that will be very valuable to the molluscan research community in general. While I believe the current state of the genome is sufficient for publication as a Data Note, I do have several suggestions for the authors to address prior to a final decision on publication,

Issues to address:

1) Citations for the references are not usually cited in the Abstract of a manuscript. I would recommend that the authors edit the Background section of the Abstract to be more general and save the specific details and citations for the main body of the manuscript.

2) On line 49, the authors state that the reads were pre-processed for bacterial sequences, duplicates and ambiguous nucleotides. I think it would be very helpful if the authors provided a little more detail on the specifics of the pre-processing, specifically with regards to the removal of likely DNA contamination since hemocyte cell isolates from hemolymph were used to generate the gDNA samples. The use of hemocytes over other tissues for the gDNA isolations could certainly contribute to significant contamination so it would be useful to know how this was specifically addressed.

3) The authors need to clarify the meaning of what they are trying to convey with the text on line 55 for a general audience. They also need to fix the typo on line 54 - an extra "f".

4) The authors misspell Crassostrea gigas both on line 69 and in Table 5.

5) The authors need to provide more details on the approach that they used to manually curate the consensus gene annotation discussed on line 81.

6) There are formatting and spacing issues for many of the tables in the pdf, although this may not be the fault of the authors and is simply a result of the pdf generator.

7) Some of the references need to be corrected. Reference #4 (Suleria et al) is listed as just accepted but it has a 2015 date. Reference #6 is not complete and contains a significant amount of

missing information. Reference #9 (Andrews) should include a link for the software program - "Reference Source, 2010" is not sufficient.

Level of Interest

Please indicate how interesting you found the manuscript: An article whose findings are important to those with closely related research interests

Quality of Written English

Please indicate the quality of language in the manuscript: Needs some language corrections before being published

Declaration of Competing Interests

Please complete a declaration of competing interests, considering the following questions:

- Have you in the past five years received reimbursements, fees, funding, or salary from an organisation that may in any way gain or lose financially from the publication of this manuscript, either now or in the future?
- Do you hold any stocks or shares in an organisation that may in any way gain or lose financially from the publication of this manuscript, either now or in the future?
- Do you hold or are you currently applying for any patents relating to the content of the manuscript?
- Have you received reimbursements, fees, funding, or salary from an organization that holds or has applied for patents relating to the content of the manuscript?
- Do you have any other financial competing interests?
- Do you have any non-financial competing interests in relation to this paper?

If you can answer no to all of the above, write 'I declare that I have no competing interests' below. If your reply is yes to any, please give details below.

I declare that I have no competing interests.

I agree to the open peer review policy of the journal. I understand that my name will be included on my report to the authors and, if the manuscript is accepted for publication, my named report including any attachments I upload will be posted on the website along with the authors' responses. I agree for my report to be made available under an Open Access Creative Commons CC-BY license (http://creativecommons.org/licenses/by/4.0/). I understand that any comments which I do not wish to be included in my named report can be included as confidential comments to the editors, which will not be published.

I agree to the open peer review policy of the journal
